# Toluene: MAK Value Documentation, addendum – Translation of the German version from 2021

**DOI:** 10.34865/mb10888e9_4ad

**Published:** 2024-12-23

**Authors:** Andrea Hartwig, MAK Commission

**Affiliations:** 1 Institute of Applied Biosciences. Department of Food Chemistry and Toxicology. Karlsruhe Institute of Technology (KIT) Adenauerring 20a, Building 50.41 76131 Karlsruhe Germany; 2 Permanent Senate Commission for the Investigation of Health Hazards of Chemical Compounds in the Work Area. Deutsche Forschungsgemeinschaft, Kennedyallee 40, 53175 Bonn, Germany. Further information: Permanent Senate Commission for the Investigation of Health Hazards of Chemical Compounds in the Work Area | DFG

**Keywords:** toluene, neurotoxicity, maximum workplace concentration, MAK value, developmental toxicity, developmental neurotoxicity, germ cell mutagenicity, toxicity, skin absorption

## Abstract

The German Commission for the Investigation of Health Hazards of Chemical Compounds in the Work Area (MAK Commission) has re-evaluated the occupational exposure limit value (maximum concentration at the workplace, MAK value), the Pregnancy Risk Group, sensitization, absorption through the skin and germ cell mutagenicity of toluene [108-88-3]. Relevant studies were identified from a literature search. The critical effects of toluene are neurotoxicity in humans, especially on the central nervous system, behavioural toxicity and ototoxicity as well as effects on colour vision. No indication of chronic effects in the range of 50 ml toluene/m^3^ were observed in an epidemiological longitudinal study in rotogravure printing, even taking into account individual estimates of lifetime exposure to toluene. Extensive, well-controlled experimental studies demonstrate no short-term toxic effects on behaviour at exposures lower than 50 ml toluene/m^3^ (in some cases even higher), which would show up as a significant reduction in performance in neuropsychological tests. Therefore, on the basis of numerous human studies, the MAK value of 50 ml/m^3^ has been confirmed. As the critical effect of toluene is systemic, the classification in Peak Limitation Category II has been retained. Since exposure peaks of 200 ml/m^3^ and simultaneous physical work had an effect on neurophysiological parameters, but not on the performance tests, the excursion factor is reduced to 2. The most sensitive end point regarding developmental toxicity and developmental neurotoxicity is the decrease in perinatal body weight. The toxicokinetic data for the inhalation of toluene and the resulting blood concentrations do not indicate significant differences between humans and animals. The margin between the LOAEC of 1000 ml/m^3^ for minor and reversible effects on the body weight of the offspring on the first postnatal day in the rat and the MAK value is sufficient. Toluene therefore remains in Pregnancy Risk Group C despite the relatively narrow, 6-fold, margin between the NOAEC for developmental toxicity and the MAK value of 50 ml/m^3^. Toluene is not genotoxic in a large number of genotoxicity tests, including dominant lethal tests. Toluene can be absorbed via the skin in toxicologically relevant amounts. The designation with “H” is therefore retained. Data in animals and in vitro show no sensitizing potential of toluene.

**Table:** 

**MAK value (1993)**	**50 ml/m^3^ (ppm) ≙ 190 mg/m^3^**
**Peak limitation (2020)**	**Category II, excursion factor 2**
	
**Absorption through the skin (1998)**	**H**
**Sensitization**	**–**
**Carcinogenicity**	**–**
**Prenatal toxicity (1993)**	**Pregnancy Risk Group C**
**Germ cell mutagenicity**	**–**
	
**BAT value (2017)** **BAT value (2009)**	**75 µg toluene/l urine** **600 µg toluene/l blood** **1.5 mg *o*-cresol/l urine (after hydrolysis)**
	
Synonyms	methylbenzene phenylmethane
Chemical name (IUPAC)	toluene
CAS number	108-88-3
Structural formula	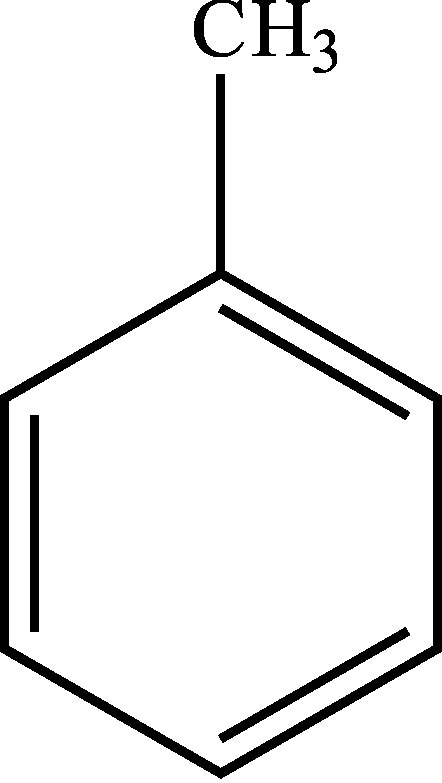
Molecular formula	C_7_H_8_
Molar mass	92.14 g/mol
Melting point	–95 °C (ECHA 2020)
Boiling point at 1013 hPa	110.6 °C (ECHA 2020)
Density at 20 °C	0.866 g/cm^3^ (ECHA 2020)
Vapour pressure at 25 °C	37.9 hPa (NCBI 2020)
log K_OW_ at 20 °C	2.73 (ECHA 2020)
Solubility at 25 °C	587 mg/l water (ECHA 2020)
**1 ml/m^3^ (ppm) ≙ 3.82 mg/m^3^**	**1 mg/m^3^ ≙ 0.262 ml/m^3^ (ppm)**
	
Hydrolytic stability	no data
Production	made from crude oil by catalytic reforming and dehydration; also a by-product of ethylene, propylene, coke and styrene production (ATSDR 2017)
Uses	solvent; starting material in the chemical and explosives industry (Greim 1996)

Documentation for toluene was published in 1986 followed by several supplements (documentation from 1986 and supplement from 1993 combined in one translation: Greim 1996; supplement from 1998: Hartwig and MAK Commission 2021 a; supplement from 2002: Hartwig and MAK Commission 2021 b). Numerous studies investigating toluene have been published since the last supplement. This addendum re-evaluates the MAK value, prenatal toxicity, germ cell mutagenicity, sensitizing effects and skin absorption.

## 1 Toxic Effects and Mode of Action

After short-term exposure, toluene induces initial neurotoxic changes like impaired well-being, drowsiness, exhaustion and headaches or which can be recorded as a reversible decline in nervous system function in the behaviour. A marked decrease in central nervous system (CNS) function is to be expected as a long-term effect. There is evidence also of sensory neurotoxicity; this was expressed as impairment of colour vision and of hearing after long-term exposure to toluene. The ototoxicity is increased by noise. Severe liver damage was induced in animal studies after exposure to toluene at high concentrations. In animal studies, reduced foetal weights, delays in growth and in foetal skeletal development were observed concurrently with maternal toxicity in the form of reduced body weights at concentrations of 1000 ml/m^3^ to 3500 ml/m^3^. A delay in incisor eruption was a postnatal effect observed in rats at 1200 ml/m^3^. No teratogenicity, embryolethality and abortive effects were induced in animals exposed to toluene up to a concentration of 3500 ml/m^3^. No effects were observed in behavioural tests with rats in offspring exposed in utero to concentrations of up to 2000 ml/m^3^. Impaired motor activity, deficits in learning and memory, an increased incidence of malformations and foetal deaths were observed in rats exposed in utero to high concentrations intended to replicate solvent abuse scenarios (8000 to 16 000 ml/m^3^, 15 to 30 minutes per day). Toluene is not genotoxic. The substance causes irritation of the skin and slight irritation of the eyes in rabbits. There is no evidence that toluene induces sensitizing effects.

## 2 Mechanism of Action

The mechanisms responsible for the acute neurotoxic effects cause the depression of CNS functions and are primarily based on reversible interactions between toluene, but not its metabolites, and the lipids and proteins of cell membranes in the CNS. In rats given toluene by intraperitoneal administration, the phosphatidylethanolamine content of isolated neuronal synapses (synaptosomes) was decreased, phospholipid methylation status was altered, as was the outer membrane fluidity, and the activity of the sodium–potassium pumps (Na^+^/K^+^–ATPase) was increased. Toluene was found to interact with voltage-gated and ligand-gated ion channels in vitro (ATSDR 2017).

Chronic exposure to high concentrations or the misuse of toluene as an intoxicant leads to structural changes in areas of the brain with high lipid content; these are detectable by magnetic resonance imaging (MRI) (Yücel et al. 2008). Additionally, neuronal apoptosis was found to be increased in the brain of rats. This led to irreversible brain damage similar to that caused by oxidative stress. An increase in the markers of oxidative stress was found also in blood samples collected from workers exposed to mixtures of solvents with a high toluene content (ATSDR 2017; Kim et al. 2011).

Acute exposure to toluene affects the synthesis, release and degradation of neurotransmitters and their binding to receptors (ATSDR 2017). Toluene is a non-competitive antagonist of the* N*-methyl-*D*-aspartate (NMDA) receptor, particularly of the NR1/2B subunit (Cruz et al. 1998). In addition to inhibiting excitatory receptors, toluene promotes the activity of the inhibitory glycine and GABA_A_ receptors (Beckstead et al. 2000). Toluene likewise affects the functioning of the nicotinic acetylcholine receptor (Bale et al. 2002; Win-Shwe et al. 2010). These mechanisms are probably the basis for the acute sedative effects of toluene. Also dopamine-dependent neurotransmission in certain areas of the brain, such as the ventral tegmental area of the midbrain and the nucleus accumbens, an integral component of the mesolimbic system, is influenced by acute exposure to toluene (Riegel et al. 2007; van Thriel 2014).

However, in animals repeatedly exposed to toluene, various subunits of the NMDA receptor were increased in the medial prefrontal cortex but not in the basal ganglia (dorsolateral striatum) (van Thriel 2014; Williams et al. 2005). A number of different animal studies demonstrated that toluene alters the expression of genes that contribute to neuroplasticity and that form the neurobiological basis of learning and memory functions (Ahmed et al. 2007; Hester et al. 2011). In summary, these mechanisms suggest that repeated exposure leads to acute sedation and effects on neuroplasticity.

The effects induced by toluene were examined in isolated Purkinje cells from male and female Long Evans rats by determining action potential output and inhibitory postsynaptic currents. Toluene (1 mM) reduced the frequency of Purkinje cell action potential output, enhanced inhibitory synaptic transmission and had no effects on the frequency of action potentials from large interneurons. These findings demonstrate that toluene affects cerebellum-dependent motor behaviour (Gmaz and McKay 2014).

Effects on the immune system were observed in other animal studies that, in turn, contribute to the effects that toluene has on the NMDA receptor and that increase the neurotoxicity induced by toluene via neuroinflammatory processes (Win-Shwe et al. 2011, 2012 a, b). Additionally, a perturbation of the hypothalamus–pituitary–adrenal axis was observed after inhalation of high concentrations of toluene (ATSDR 2017). However, the relevance of these findings for humans is unclear.

Toluene caused hearing loss in rats by directly altering the cochlear microphonic potential. It is assumed that the changes in colour vision induced by toluene are caused by the interaction of toluene with dopaminergic mechanisms of retinal cells or toxic demyelination of optic nerve fibres (ATSDR 2017).

The effects induced by toluene in concentrations of 100 and 200 µM were examined in vitro using outer hair cells isolated from the cochlea and the spiral ganglion cells of guinea pigs. Toluene led to a dose-dependent shortening of the outer hair cells. At concentrations of 30 µM and above, toluene increased the intracellular calcium levels of both outer hair cells and spiral ganglion cells (Liu and Fechter 1997).

## 3 Toxicokinetics and Metabolism

### 3.1 Absorption, distribution and elimination

Blood toluene levels of 10 µmol/l (0.92 mg/l) were determined in samples taken directly after the exposure of 9 male volunteers to a toluene concentration of 200 mg/m^3^ (about 52 ml/m^3^) for 2 hours while exercising at 50 watts on a bicycle ergometer. The steady state was almost reached after an hour. On average, about 50% of the inhaled amount was absorbed. The elimination of toluene from the blood was triphasic. The half-life of the alpha phase was 3 minutes, that of the beta phase 40 minutes and that of the terminal phase 738 minutes. Determinations taken 4 and 20 hours after the end of exposure found that 65% and 78%, respectively, of the amount absorbed had been excreted with the urine as hippuric acid (Löf et al. 1993).

Toluene has a mean blood:air partition coefficient of 15.11 (Meulenberg and Vijverberg 2000).

Several physiologically based pharmacokinetic (PBPK) models were described in ATSDR (2017). Two studies carried out by the same research group examined the concentrations in the blood after inhalation exposure of humans and rats. After exposure to toluene for 3 to 4 hours at the same concentration of 100 ml/m^3^, slightly higher concentrations of toluene were found in the blood of rats in comparison with the levels found in humans (2–3 mg/l in rats, about 1 mg/l in humans) (Benignus et al. 2006; Kenyon et al. 2008). After exposure of Wistar rats by inhalation for 4 hours at a concentration of 125 ml/m^3^, the concentration in the blood was determined to be 2 mg/l (Kishi et al. 1988).

In rats, therefore, a higher concentration of toluene is reached in the blood at the same external concentration compared to humans.

Oral absorption is assumed to be 100% in rats (Turkall et al. 1991). After inhalation exposure, humans absorbed 50% of the administered amount (see above: Löf et al. 1993).

### 3.2 Metabolism

Toluene is metabolized mainly by oxidative pathways to form benzoic acid, which is excreted with the urine as hippuric acid. Also trace amounts of benzoyl glucuronide, the sulfate and glucuronide conjugates of *o*-cresol and *p*-cresol, *S*-benzylmercapturic acid and *S*-*p*-toluylmercapturic acid were found (ATSDR 2017).

## 4 Effects in Humans

### 4.1 Single exposures

A PBPK model was applied to the findings of different experimental studies investigating exposure in healthy test persons. The PBPK model was used on the acute exposure data from 6 different studies to model the toluene concentrations in the arterial blood and to compare the toxic effects on behaviour on the basis of this exposure marker. The modelling carried out by the authors demonstrated that the choice reaction time was decreased by 10% at an arterial blood concentration of toluene of 3 ml/l. On the basis of this concentration in the blood, the authors extrapolated the corresponding exposure durations and concentrations and predicted that exposure for 2 hours to a toluene concentration of 27 ml/m^3^ would lead to such a decrease in performance caused by behavioural toxicity (Benignus et al. 1998). The analysis contains a number of uncertainties in the mathematical models and in the summary of the cognitive performance tests carried out by the individual studies. Therefore, the data are not suitable for the derivation of a MAK value.

In another meta-analysis carried out by the same authors, the effects of toluene estimated in this manner were compared with the toxic effects on behaviour induced by ethanol. The concentrations in the blood were determined by a GPAT model (general physiological and toxicokinetic model). The dose-equivalent function shows that in venous blood, 3 mg toluene/l corresponds to an ethanol blood concentration of approx. 1 g/l, which, according to the model, would increase the choice reaction time by about 14% with this data set. The authors then extrapolated the level and duration of exposure from these data. They calculated that the effects induced by exposure to a toluene concentration of 100 ml/m^3^ at rest for a period of 8 hours are equivalent to the effects induced by a blood ethanol level of about 0.62 g/l. If physical exercise at 50 watts were added to the model, the toluene concentration in the blood would be equivalent to a blood ethanol level of 1.27 g/l. At this blood ethanol level, the model applied by the authors predicts a 20% increase in choice reaction time. At a concentration of 50 ml/m^3^ and physical exercise at 50 watts, the choice reaction time is expected to increase by less than 8% which should correspond to the effects induced by a blood ethanol level of 0.7 g/l (Benignus et al. 2005). However, two of the figures in the publication show that a toluene concentration of 1 mg/l is equivalent to an increase in choice reaction time of about 3% and a blood ethanol level of 0.5 g/l. A study with test persons found that exposure to a toluene concentration of 50 ml/m^3^ at a workload of 50 watts was equivalent to a blood toluene level of 1 mg/l (see Löf et al. 1993). Therefore, the GPAT model overestimates the effect size of toluene. At a concentration of 50 ml/m^3^ and a workload of 50 watts, the choice reaction time is expected to increase by 3%; this is equivalent to the effects induced by a blood ethanol level of 0.5 g/l. Again, this study is not suitable for the derivation of a MAK value due to the uncertainties involved with the models.

A group of 20 persons was exposed to a toluene concentration of 50 ml/m^3^ for 4.5 hours. No signs of drowsiness were determined by a pupillographic test. Acute symptoms in various areas were evaluated by questionnaire. Only the occurrence of the individual symptoms “unpleasant smell” and “throat irritation” was increased with statistical significance; the subjective reports of tiredness were not increased (Muttray et al. 2005).

A group of 8 print shop workers underwent colour discrimination tests before and after carrying out cleaning activities with toluene. Toluene was the only solvent applied. The blood toluene levels were between 3.61 and 7.37 mg/l after cleaning (BAT value: 0.6 mg/l). Toluene had no effect on the colour vision of the exposed persons in this study (Muttray et al. 1999).

Seventeen healthy test persons were examined for neurobehavioural and neurophysiological effects after a single exposure to toluene at a concentration of 200 ml/m^3^ for 40 minutes with intermittent exercise on a bicycle ergometer for 15 minutes in total. The test persons underwent behavioural testing once before and twice after the exposure period of 40 minutes, engaging in tasks designed to evaluate different parameters of visual attention. The number of incorrect responses in the test was increased only in the group of test persons with exposure to toluene in the tests carried out after the exposure to toluene. The differences in trend were observed during a task that required the inhibition of a response. Overall, the test persons in the toluene group responded slower after exposure in this test than the control persons. In addition, the neurophysiological findings (N1 amplitude in the EEG) demonstrated that visual stimuli were processed less efficiently (Kobald et al. 2015).

Another experimental study with exposure of 17 healthy test persons investigated the effects of peak exposures on neurophysiological processes and motor learning. The test persons were exposed during 4 hours to two 35-minute periods of peak exposure to 200 ml/m^3^ while performing controlled physical exercise on a bicycle ergometer. The 17 test persons completed another 4-hour session during which they were not exposed to toluene. The acute effects of toluene on neurophysiological parameters were investigated by transcranial direct current stimulation (tDCS), a non-invasive technique to induce neuroplasticity. The learning efficiency of the test persons was evaluated using a serial reaction time task. The outcomes of the learning tasks remained unaffected by exposure to toluene. Neuroplasticity was induced by tDCS under control conditions; however, this neurophysiological effect was not observed after exposure to toluene (Yavari et al. 2018). As the NMDA receptor plays an important role in inducing neuroplasticity, its inhibition may offer a mechanistic explanation for this finding.

Exposure to a toluene concentration of 15 ml/m^3^ for 15 minutes impaired short-term and long-term memory function and psychomotor coordination in patients with chemical sensitivity (Little et al. 1999).

### 4.2 Repeated exposure

#### 4.2.1 Neurotoxicity

Workers (n = 98) of a rotogravure plant were examined neuropsychologically using a cognitive function scanner and underwent a neurological evaluation to assess a total of 17 performance variables concerning coordination ability, tremor and position stability. Questionnaires were used to collect information on the occurrence of symptoms such as headache, dizziness and reduced memory function. Three groups were established based on the duration of exposure: Group 0 (no exposure, 19 persons), Group 1 (1–12 years, 30 persons), Group 2 (> 12 years, 49 persons). Of the workers assigned to Group 2, 37 had been exposed to a concentration of 100 ml/m^3^ for longer than 10 years; all others had been exposed to a toluene concentration < 20 ml/m^3^. In the group of workers exposed to a toluene concentration < 20 ml/m^3^, the findings in the workers who were exposed for less than 13 years did not differ from those in the control persons. However, statistically significant differences between the findings in the controls (Group 0) and the workers with long-term or high exposure levels (Group 2) were found for symptom experience and 2 of the 17 performance variables. Toluene was the only organic solvent used in the rotogravure printing process (Eller et al. 1999). The relevance of this study is limited because of insufficient consideration of covariables and confounders (age and alcohol use). Also, the authors assume that there was a certain degree of error in the estimation of past exposure levels, as the exposure concentrations up to 1983 were higher.

In a study investigating 72 workers who were employed for at least 5 years at a printing company or a pathology laboratory with exposure only to toluene (9 to 467 ml/m^3^), no changes in cognitive and neurological functions were determined in comparison with the findings in control persons. Also the results of the psychometric tests did not differ from those determined in control persons. However, exposed persons reported irritation of the mucous membranes with greater frequency than control persons; the increase was statistically significant. The tests were carried out at least 2 days after the end of exposure to ensure that toluene had been eliminated from the blood (Deschamps et al. 2001).

A cross-sectional study assigned 278 workers from different rotogravure plants to 2 exposure groups. The workers had a mean age of 39.8 years and had been exposed to toluene for a mean duration of 14.9 years. In the first group (n = 154), the mean lifetime weighted average exposure (LWAE) was 45.1 ml/m^3^ with a mean current exposure of 24.7 ml/m^3^. In the second group (n = 124), the LWAE was 9.3 ml/m^3^ with a mean current exposure of 3.3 ml/m^3^. Manual dexterity was tested using a motor performance test battery (Schuhfried motor performance series). The subjective symptoms were compiled by psychological-neurological questionnaire. Both groups were found to have similar fine motor skills. No statistically significant differences were found between the exposed persons and the control persons. An analysis of the symptoms did not reveal any differences between the 2 groups (Zupanic et al. 2002).

To investigate possible effects induced by exposure to low concentrations of toluene, another cross-sectional study examined 129 workers chosen from among 110 workers of Plant A and 252 workers of Plant B who carried out offset and heliogravure printing processes at two different printing plants. Toluene concentrations at both plants were in the range from 0 to 18 ml/m^3^ at Plant A (offset) and from 2 to 27 ml/m^3^ at Plant B (heliogravure). At Plant A, individual samples were taken to determine the toluene concentrations in the breathing zone of each worker. At Plant B, individual samples were not taken for each worker, but representative samples were taken in each work area to individually estimate current exposure levels. Past levels of exposure to toluene were estimated to have been between 0 and 179 ml/m^3^. The duration of exposure was between 0 and 40 years with a mean length of 14 years. The individual cumulative exposure indices (CEI) were between 0 and 2352 ml/m^3^ × years. The neurotoxic symptoms were ascertained by EUROQUEST questionnaire and any toxic effects on behaviour were determined by standardized test methods (neurobehavioural evaluation system). The following 6 tests were performed: simple reaction time, digit symbol test, digit span forwards and backwards, visual short-term memory and tests for associative learning that examined learning and memory aptitude. Nine parameters were derived from these tests and evaluated by regression analysis after adjustment for covariables. The CEI and the current exposure levels of the workers were used as estimators of individual exposure levels. A statistically significant association was found only between the CEI and test performance; no associations were found between the CEI and the reported symptoms. When the determined/estimated levels of current exposure to toluene were applied to a model, concentration-dependent decreases in performance were observed in both versions of the digit span test and therefore in short-term memory. The authors compared the effects of toluene exposure and age on test performance. In the digit span forwards, performance was decreased by 1 digit for 25 years of life. In the regression model, a corresponding decrease in performance was observed at current levels of exposure to toluene above 36 ml/m^3^. In the more difficult version, the digit span backwards, 14 additional years of life were necessary to reduce performance by a “memorized” digit. The authors found comparable effects at current levels of exposure to toluene above 26 ml/m^3^. Although memory performance was assessed by a number of other tests, this parameter did not reveal any concentration-dependent effects. The authors assumed that alcohol consumption was highly underestimated by this study. In addition, they drew attention to three shortcomings of the study: 1) insufficient statistical power, 2) imprecise data for past exposure levels and 3) a possible reversibility of toluene neurotoxicity by reducing actual exposure levels (Chouanière et al. 2002). Overall, this cross-sectional study has a number of methodological shortcomings, particularly in the estimation of exposure and the adjustment for multiple comparisons in the regression models.

A meta-analysis reviewed the data from 10 epidemiological studies to determine the toxic effects on behaviour induced by occupational exposure to toluene. Data for 408 control persons and 447 exposed persons were analysed. Standardized effect sizes were calculated for 6 test variables (digit span forwards/backwards, digit symbol test (paper or computer formats), simple reaction time, block design) to evaluate the mean effect size estimator for significance according to the random effects model. The mean exposure concentration in the 10 studies was 57 ml/m^3^ (20–117 ml/m^3^). Although several studies reported a decrease in performance in neuropsychological tests among the exposed persons, a systematic review did not find toxic effects on behaviour at mean estimated exposure levels of up to 90 ml/m^3^. However, the author suggested that the study groups needed to be homogenized with respect to intelligence, cultural background and practice trials in order to be able to compare the findings of the studies and combine them quantitatively within a meta-analysis (Meyer-Baron 2005). Also, the individual studies need to have a more standardized exposure determination, to be able to derive concentration–effect relationships.

A study investigated 54 workers at different plants who were occupationally exposed to toluene for toxic effects on behaviour using a standardized test battery. The tests applied were finger tapping, digit span forwards and backwards, simple reaction time, selective attention and the digit symbol test. The “low” exposure group was exposed to toluene concentrations < 10 ml/m^3^, the “medium” exposure group to 20–30 ml/m^3^ and the “high” exposure group to 70–80 ml/m^3^. The data were adjusted for age, education and work duration by means of covariance analyses. Decreased performance in the tests digit span forwards, finger tapping and selective attention was found only in the “high” exposure group (Kang et al. 2005). Therefore, the effects were observed only at concentrations above the MAK value.

The MRI results of a 38-year-old woman with chronic headaches and nausea revealed a T2-hyperintense lesion in the cerebral white matter of the left frontoparietal lobe. The woman worked at a plant in which a paint thinner containing about 60% toluene was used. The other constituents of the paint thinner were xylene, ethyl acetate and butyl acetate (Kobayashi 2014).

#### 4.2.2 Hearing loss

A group of 190 printing workers (toluene concentrations: 75–365 ml/m^3^, exposure period: 6 years, 88–98 dB(A)) was examined by regression analysis to determine which exposure conditions are associated with bilateral hearing loss of more than 25 dB. Bilateral hearing loss was most strongly associated with concurrent exposure to both toluene and noise in comparison with the degree of hearing loss found after exposure only to toluene or only to noise (Morata et al. 1993). In a follow-up study of 124 persons with exposure to toluene, a statistically significant relationship was found between the concentration of hippuric acid in the urine and bilateral hearing loss of more than 25 dB. An odds ratio (OR) > 2 was determined by back calculation from the hippuric acid concentration to the toluene concentration at the workplace at 50 ml/m^3^. However, the exposed persons were concurrently exposed to toluene, ethyl acetate and ethanol (Morata et al. 1997).

Simultaneous exposure to solvents and noise has been shown to have a greater impact on hearing loss than exposure to noise alone. A study determined an OR of 5.3 (95% confidence interval (CI): 2.6–10.9) for hearing loss for a group of 96 exposed persons (toluene and *n*-hexane, individual average worklife exposure index 1.6 ± 1.1) who were concurrently exposed to noise (> 85 dB) (Sliwinska-Kowalska et al. 2005).

#### 4.2.3 Colour vision

A study of 33 workers in the rubber industry with exposure to toluene and 16 workers without exposure found that the ability to discriminate colours was impaired after repeated exposure to toluene. To determine the level of exposure, unmetabolized toluene was analysed in the urine before and after the work shift. The mean value was found to be 63 ± 27 µg/l urine, which is equivalent to a toluene concentration in the air of about 42 ml/m^3^. To calculate cumulative exposure levels, the toluene concentration in the urine was multiplied by the duration of exposure. The mean duration of past exposure was less than or equal to 10 years. The colour vision of the test persons was evaluated by means of the Lanthony D-15d test (with desaturated colours), a colour arrangement test. Two indicators were derived, the Color Confusion Index (CCI) and Total Confusion Index (TOTCI). Both indices are based on deviations from the correct spectral arrangement of 15 colours. However, the TOTCI uses a different formula to calculate the deviation from 1.00, the value that corresponds to the error-free arrangement of the colour pans. A mean CCI of 1.29 was determined for exposed persons and 1.10 for the controls, the TOTCI was 1.49 for exposed persons and 1.16 for the controls. The differences determined for both indices were statistically significant. In addition, a positive correlation was found between the two indices and the cumulative toluene dose. The correlation with the TOTCI was slightly higher. Although the workers were exposed to a mixture of different solvents in the adhesive, the authors explained that 1) toluene was the main constituent of the adhesive and 2) the other solvents in this mixture are not expected to have an effect on colour vision (Cavalleri et al. 2000). However, the findings do not appear to have been adjusted for possible co-factors, although the workers exposed to toluene consumed more alcohol than the control persons. The authors reported that the impairments in colour vision were subclinical and the exposed persons did not actually notice this impairment. However, at only 10 years the duration of exposure was somewhat short in comparison with a working lifetime of 40 years. The concentration of 42 ml/m^3^ can be regarded as the LOAEC (lowest observed adverse effect concentration); however, any attempt to interpret the data needs to consider the lack of adjustment for the covariable alcohol consumption.

A study in 92 persons with exposure to toluene found impairment of the colour discrimination ability. This dyschromatopsia was observed both before and after adjusting for age and alcohol consumption. Exposure was to a mean toluene concentration of 136 (50–296) mg/m^3^ (36 (13–78) ml/m^3^) (Campagna et al. 2001).

A longitudinal study investigated possible effects of occupational exposure to toluene on colour vision over a period of 4 years. Initially, a total of 278 persons with exposure took part in the study; the number of participants was decreased to 241 at the second examination and to 216 workers at the third examination. Data for all 3 examinations were available for a total of 162 exposed persons. The printing workers in the “high” exposure group were found to have current exposure levels of 26 ± 21 ml/m^3^ and those in the “low” exposure group were exposed to 3 ± 4 ml/m^3^. The LWAE was 43 ml/m^3^ and 9 ml/m^3^, respectively. Colour vision was evaluated by means of the Lanthony D-15d test and the CCI was determined. A deviation from 1 in the CCI indicates an impairment of colour discrimination on the blue–yellow axis. Multiple regression analyses and repeated analyses of variance did not reveal statistically significant effects after exposure to toluene (Schäper et al. 2004). However, age and professional qualifications were found to have statistically significant effects; many of the other epidemiological studies did not take their impact into consideration.

A study investigated 41 of 46 workers with low levels of exposure (11.30 to 49.30 ml toluene/m^3^) and 32 of 37 workers with higher levels of exposure (66 to 250 ml toluene/m^3^). The control group consisted of 83 persons without exposure. The study likewise applied the Lanthony D-15d test; the evaluation was based on errors along the blue–yellow and red–green axes of colour vision. The number of persons with dyschromatopsia, particularly along the blue–yellow axis (type III), was increased with statistical significance in the group with higher levels of exposure in comparison with the incidence found in the control group. The findings in the group with low levels of exposure did not differ with statistical significance from the findings in the control group, but did differ with statistical significance from the findings in the group with higher levels of exposure (Zavalić et al. 1998). Evidence of colour vision impairment was found in this study only at levels above the MAK value.

A meta-analysis of 15 studies chosen from 39 published studies systematically reviewed the effects of exposure to solvents on colour vision. However, only 4 studies of toluene provided the data necessary for a quantitative summary of the findings. In these studies, the mean toluene concentrations were 26, 32, 42 and 50 ml/m^3^ and the mean duration of exposure was between 9 and 16 years. Colour vision was evaluated by means of the Lanthony D-15d test. A CCI of 1.07 to 1.29 was determined for the exposed persons and of 1.10 to 1.19 for the control persons. The effect sizes in these studies were between –0.34 and 2.02. In the meta-analysis, the mean effect size of 0.15 determined by applying a random effects model did not differ from 0 with statistical significance. Therefore, the findings in the exposed persons did not differ with statistical significance from those in the control persons. However, the exposure concentrations in all studies were relatively low and the different studies did not take confounding factors such as age, sex, alcohol consumption and smoking habits adequately into consideration (Paramei et al. 2004).

A high correlation between exposure to high toluene concentrations at the workplace and dyschromatopsia was found by Muttray et al. (2019). A total of 51 workers exposed to toluene and 51 control persons were evaluated by means of the Farnsworth panel D-15 test, Lanthony D-15 test, Velhagen plate test and the Standard Pseudoisochromatic Plates. The toluene concentration in the blood of the exposed persons was 1.59 mg/l. The time-weighted average concentration of toluene in the air was 97 to 92 ml/m^3^. Exposed persons demonstrated slight deficiencies in colour vision at concentrations that were considerably higher than the MAK value.

### 4.3 Local effects on skin and mucous membranes

Toluene degreases and dries out the skin (Greim 1996). Repeated skin contact may lead to toxic contact dermatitis (ECHA 2020; EU 2003).

Irritation of the eyes and nose was found in 16 male volunteers who were exposed to a toluene concentration of 100 ml/m^3^ for 6 hours a day on 4 consecutive days. No irritation of the eyes or nose was reported in a questionnaire after exposure to a concentration of 40 ml/m^3^ (Greim 1996).

In another study with test persons, neither eye irritation nor watery eyes or blurred vision were induced in 20 male volunteers after exposure to a toluene concentration of 50 ml/m^3^ for 4.5 hours (Muttray et al. 2005).

### 4.4 Allergenic effects

There are no data available.

### 4.5 Reproductive and developmental toxicity

A comprehensive review of the studies that investigated reproductive toxicity is included in the toxicological profile for toluene published by the Agency for Toxic Substances and Disease Registry (ATSDR 2017). The most important studies are summarized below.

#### 4.5.1 Fertility

The fecundity (likelihood of conception) of female, but not of male workers in German printing companies was decreased during periods of occupational exposure to toluene in comparison with the values determined during periods of employment without exposure to toluene. Three groups were formed based on occupational history and the levels of exposure determined in previous years (determinations were carried out by industrial hygienists of the employer’s liability insurance company): low exposure (for example, stacking and bookbinding; < 10 ml/m^3^), medium exposure (preparation of cylinders, galvanisers; 10–30 ml/m^3^) and high exposure (printers; < 200 ml/m^3^ prior to 1984, < 100 ml/m^3^ from 1984–1994 and < 50 ml/m^3^ after 1994). The data relating to the reproductive and occupational history of the workers were analysed to determine a fecundity index. This was based on the time from the beginning of unprotected intercourse to conception during periods of occupational exposure and during those without exposure. The data were adjusted for age, ethnicity, smoking, parity, pelvic inflammatory diseases and frequency of sexual intercourse. In the women, the fecundity index was reduced with statistical significance in the periods with exposure to toluene (0.47; 95% CI: 0.29–0.77); no effects on fecundity were observed in male workers and their partners in the periods with exposure to toluene. In this study, women were employed only in work areas of the printing companies with expectedly low toluene concentrations in the air (stacking and bookbinding) and not in areas with high (operating printing machines) and medium (preparation of cylinders) exposure to toluene. The men worked in all 3 areas (Plenge-Bönig and Karmaus 1999).

In summary, the available data do not provide reliable evidence that acute or repeated inhalation of toluene leads to reproductive toxicity in humans. There is weak evidence linking occupational exposure to toluene to an increased incidence of spontaneous abortions (ATSDR 2017; Greim 1996) and reduced fecundity of female workers (Plenge-Bönig and Karmaus 1999). A population-based cohort study found an increased risk of premature births with increasing levels of environmental exposure to toluene (ATSDR 2017); however, co-exposure to numerous other pollutants (that were not accounted for in the statistical analysis) limits the validity of the conclusions drawn from the study findings (ATSDR 2017).

#### 4.5.2 Developmental toxicity

The symptoms induced by toluene in children whose mothers inhaled high concentrations (4000–12 000 ml/m^3^) of toluene or other organic solvents during pregnancy are similar to those associated with the foetal alcohol syndrome resulting from exposure to ethanol (ATSDR 2017). The findings in humans are not suitable for a quantitative assessment as there are no data for the duration of exposure and a mixture of substances was involved (Greim 1996).

There is only one study available that investigated exposure to low concentrations at the workplace. The findings of a retrospective study with 14 women in Finland who were exposed to a mixture of different solvents suggest that exposure may increase the risk of central nervous system anomalies and neural tube defects (ATSDR 2017). As a result of the small number of cases investigated and the exposure to a mixture of solvents, this study cannot be used to draw conclusions about developmental toxicity in humans after exposure to low levels of toluene.

### 4.6 Genotoxicity

Several of the studies were reviewed in the documentation published in 1986 (Greim 1996); these are included again below for comparative purposes.

A number of studies investigated the genotoxic potential of toluene in workers of printing companies who were exposed primarily to toluene at the workplace. However, only a small number of persons were studied, they were exposed to a mixture of substances and the findings were contradictory.

The incidence of DNA strand breaks (comet assay) was found to be increased both in the lymphocytes of the blood of painters (Moro et al. 2012) and in those of shoe factory workers (Heuser et al. 2005, 2007). However, the studies did not provide data for the toluene concentrations. The painters were occupationally exposed for an average of 46.15 ± 9.94 months; the hippuric acid concentrations in the urine were below 1.60 g/g creatinine. At the shoe factory, the duration of exposure was, on average, 5.80 ± 4.03 years for workers who handled water-based adhesives and 3.98 ± 4.13 years for workers who handled solvent-based adhesives. The workers were exposed to a mixture of substances including hexane, acetone, 2-butanone, polyurethane and polychloroprene. No data were provided for the toluene concentrations in the air.

By contrast, no exposure-related differences in DNA damage in leukocytes were determined by the comet assay in female workers of a shoe factory who were exposed to toluene concentrations of 28 to 121 ml/m^3^ (Pitarque et al. 1999).

The incidence of structural chromosomal aberrations was increased in the blood lymphocytes of 20 male rotogravure printers in comparison with the incidence found in 24 control persons without exposure. The rotogravure printers were exposed for at least 16 years by inhalation to toluene concentrations of 200 to 300 ml/m^3^ (< 0.3% benzene) and by absorption via the skin. In particular, the incidence of chromatid breaks and sister chromatid exchange (SCE) was increased in workers with exposure to toluene, both in smokers and in non-smokers. The authors concluded that toluene induces weak genotoxic effects in this concentration range (Bauchinger et al. 1982).

In 42 male printing workers who were exposed to toluene at the workplace for an average of 19 years, the incidence of SCE determined in the lymphocytes was about 3 times as high as that found in the control group (n = 45 ♂). A statistically significant correlation was established between the frequency of SCE and the total cresol to hippuric acid ratio in the urine of printing workers after correcting for smoking status. On the other hand, no statistically significant relationships were determined with *o*-cresol, *p*-cresol or hippuric acid alone. The concentrations in the ambient air fluctuated between 37 and 86 ml/m^3^ (median: 62 ml/m^3^). Individual exposure to toluene, as determined by portable detectors, was found to be between 15 and 118 ml/m^3^ (median: 60 ml/m^3^). There was only slight contamination with benzene (0.01%) and xylene (0.165%) (Hammer 2002; Hammer et al. 1998).

In 14 workers of a rotoprinting factory (duration of exposure: 1.5–26 years) who were exposed to toluene concentrations in the range from 100 to 200 ml/m^3^ (exposure peaks between 500–700 ml/m^3^), the incidence of chromosomal breaks in the blood lymphocytes was increased in comparison with that of the control group. However, the increased incidence did not correlate with the level of exposure (Funes-Cravioto et al. 1977).

The role of exposure to toluene in the induction of genotoxic effects was investigated in the peripheral lymphocytes of 21 printing workers who were exposed to toluene for an average of 25 years (range: 0.5–37) and compared with the findings in a control group without exposure (n = 21; blood toluene levels: ≤ 0.1 µmol/l). Daily determinations taken over 1 week revealed that the workers were exposed to a mean toluene concentration of 39 ml/m^3^ per week (range: 8–110 ml/m^3^ in the air; blood toluene level: 1.6 µmol/l). In the analysis of genotoxic potential, the micronucleus frequency was found to be affected by the mitogen selected to stimulate the growth of the cultured lymphocytes. In comparison with the levels determined in the control group, the frequency of micronuclei was increased with statistical significance (p = 0.03) in the pokeweed mitogen (PWM)-stimulated lymphocytes (activation of T-cells and B-cells) of printing workers, but not in the phytohemagglutinin mitogen (PHA)-stimulated lymphocytes (only T-cell activation). The frequency of small micronuclei (size ratio micronuclei/nucleus ≤ 0.03) in PWM-stimulated lymphocytes correlated positively with exposure to toluene (p = 0.05) and with the blood toluene levels of the printing workers (p = 0.0005). In PHA-stimulated lymphocytes, no correlation was found between exposure parameters and the frequency of small micronuclei, but a weak association was determined between the cumulative exposure index (sum of exposure period (years) × corresponding estimates of exposure (mg/m^3^)) of all workers and the number of total micronuclei (p = 0.07). Chromosomal aberrations (chromosomal breaks) in PHA-stimulated lymphocytes were associated with past exposure to benzene (0.03%/year; p = 0.01). Smoking status and age were taken into consideration for the calculation of statistical significance (Nise et al. 1991).

In 23 printers who were occupationally exposed to toluene concentrations of 155 ml/m^3^ (range: 8–410 ml/m^3^) for an average duration of 5 years, a statistically significant increase (p = 0.05) in the number of chromosomal aberrations was found in the peripheral lymphocytes and correspondingly more (p = 0.01) hippuric acid in the urine in comparison with the levels determined in the control group (n = 22) (Pelclová et al. 2000).

Another study that investigated 42 printers who were exposed to workplace concentrations in the range from 104 to 1170 ml/m^3^ for an average of 13 years and 28 technical employees and office workers who were exposed to toluene concentrations of 2.1 to 4.3 ml/m^3^ and additionally spent 0 to 2 hours per day in the rotogravure workshop found that the incidence of chromosomal aberrations, but not of SCE, was increased in the lymphocytes with statistical significance in comparison with the levels determined in the control group (n = 32) (Pelclová et al. 1990). Structural aberrations (mainly chromatid breaks) were found in 3.64% ± 2.05% of cells in the printers, in 3.32% ± 1.63% of cells in the group with low levels of exposure and in 2.09% ± 1.53% of cells in the control group. The hippuric acid levels in the urine were 6.31 ± 3.41 mmol/l in the control group, 12.89 ± 4.64 mmol/l in the group with low exposure and 38.28 ± 17.53 mmol/l in the printers. The blood toluene levels were 10.3 ± 3.1 and 124.0 ± 63.1, respectively, in the latter 2 groups. The toluene used for the tests was found to have a purity of 98.4%; the impurities it contained were 0.45% ethylbenzene, 0.4% *m*-xylene and *p*-xylene, and 0.3% *o*-xylene.

In comparison with the levels determined in the control groups without exposure, the increased levels of chromosomal aberrations, but not of SCE, in the peripheral lymphocytes of 27 former printers who had been exposed to toluene concentrations of 200 to 300 ml/m^3^ for 1 to 34 years remained elevated for up to 2 years after the end of employment (Schmid et al. 1985).

The incidence (p < 0.05) of SCE (4.81 ± 0.92) was higher in male painters (n = 30) in comparison with the incidence found in 30 control persons (1.73 ± 0.54). The toluene concentrations in the air were not determined; the average hippuric acid level in the urine was 2.5 times as high as the level found in the control group. Higher frequencies of SCE were found in test persons with specific polymorphisms in cytochrome P450 (CYP) 2E1 and CYP1A1, the enzymes responsible for the metabolism of toluene, in comparison with the frequencies determined in exposed persons with the wild type genotype (Priya et al. 2015).

In general, there has been discussion whether the positive findings in the workers of printing companies reported by several studies are attributable to substances other than toluene, such as polycyclic aromatic hydrocarbons from carbon black in printing inks (Hammer et al. 1998; Pelclová et al. 1990, 2000). However, printing inks were not found to be mutagenic in mutagenicity tests in Salmonella typhimurium (Pelclová et al. 2000).

After exposure to toluene (concentrations unknown) for 14 years, the incidence of dicentric chromosomes was found to be increased in 31 male printers in comparison with the incidence determined in the control group (n = 31 ♂). No statistically significant differences in chromatid aberrations and SCE were found (Carbonell et al. 1982).

In 24 male rotary printing workers exposed to a toluene concentration of 200 ml/m^3^ for an average of 3 to 15 years with intermittent periods of short-term exposure to much higher levels, the incidence of chromosomal aberrations was not increased in comparison with the incidence found in the control group consisting of 34 male persons of the same age (matched pairs) (Forni 1971).

Likewise, the incidences of SCE or chromosomal aberrations found after exposure to toluene in the concentration range from 7 to 112 ml/m^3^ (n = 32 ♂, 8 hours/day, exposure for 3–35 years) did not differ from the incidences determined in the control group (n = 15 ♂). The incidence of SCE was higher with statistical significance in smokers in comparison with the incidence determined in non-smokers. The benzene content in toluene was regularly monitored by gas chromatography; it was below 0.05% at all-time points and on average 0.006% (Mäki-Paakkanen et al. 1980).

Studies of the genotoxic effects induced by toluene that investigated workers from other industrial sectors likewise reported inconsistent findings. In a study with 16 male paint industry workers who were exposed for more than 10 years (8 hours/day) to a mixture of solvents (11 substances, for example xylene, ethanol) in the air with a mean toluene concentration of 2.9 ml/m^3^ (0.3–329 ml/m^3^), the incidences of SCE and chromosomal aberrations in the exposed workers did not differ with statistical significance from those in the control group (n = 17; matched pairs) (Haglund et al. 1980).

In 52 female workers of a shoe factory who were exposed to a mixture of toluene (20 or 63 ml/m^3^), benzene, acetone and other solvents, the frequency of micronuclei, but not that of SCE, in cultured lymphocytes was increased with statistical significance in comparison with the frequencies determined in workers without exposure. Hippuric acid was determined in the urine as a biomarker of toluene exposure (Pitarque et al. 2002).

In 34 workers (n = 11 ♀ / 23 ♂) of a shoe factory who were exposed to a concentration of 8 ml/m^3^ (1.7–95 ml/m^3^) for an average of 10 years (0.3–46), the frequency of micronuclei was increased in comparison with the frequency determined in the control persons. In a multivariate analysis including age, BMI, smoking habits, alcohol consumption, and the duration and level of exposure to acetone, ethyl acetate, 2-butanone and toluene, the toluene concentration was the only variable that had statistically significant effects on the induction of micronuclei (González-Yebra et al. 2009).

Two other studies did not find a correlation between exposure to solvent-based adhesives (n = 29 ♂; exposed for 3.98 ± 4.13 years) (Heuser et al. 2005) or paints and the induction of micronuclei, either in lymphocytes or in the cells of the buccal mucosa (Heuser et al. 2005, 2007; Moro et al. 2012).

In a solvent abuse study, DNA damage determined by the alkaline comet assay in the peripheral blood lymphocytes of chronic glue sniffers (n = 20 ♂; 16.2 ± 2.8 years) was increased with statistical significance in comparison with the levels determined in age-matched controls (n = 20), irrespective of smoking status. The exposure concentrations were not given; most of the adhesives used contained high levels of toluene in addition to other volatile components such as acetone, ethyl acetate and 2-butanone. The mean values for the excretion of hippuric acid and *o*-cresol were 73 and 1582-fold higher in glue sniffers in comparison with the values reported for the historical controls (n = 54) (Cok et al. 2004).

In a controlled exposure study, 5 male test persons (non-smokers) were exposed by inhalation in an exposure chamber for 7 hours per day over 3 consecutive days to toluene concentrations of 50 ml/m^3^. This was repeated 3 times at intervals of 2 weeks. No effects on the frequency of SCE were detected in blood samples taken before and after each 3-day period of exposure (Richer et al. 1993).

**Summary**: The evaluated studies of workers in printing companies with occupational exposure mainly to toluene reported in part contradictory findings with regard to the genotoxic potential of toluene. The number of persons examined was small and exposure was to a mixture of substances. Toluene did not induce SCE in 5 male test persons after controlled exposure at the level of the MAK value of 50 ml/m^3^.

## 5 Animal Experiments and in vitro Studies

### 5.1 Acute toxicity

#### 5.1.1 Inhalation

In rats anaesthetized and mechanically ventilated for 10 minutes (no other details, 5 per group) with toluene concentrations between 18 and 450 ml/m^3^, increased vascular permeability was found in the main bronchi (at toluene concentrations of 30 ml/m^3^ and above) and in the trachea (at 50 ml/m^3^ and above) (Sakamoto et al. 2012).

In male pigmented DA/HAN rats (10/group) exposed to toluene concentrations of 0 or 100 ml/m^3^ for 4 hours, the eye position at rest was unsteady, the eye velocity was slower and eye movements were more irregular in response to visual stimulation at the end of exposure; these effects persisted after termination of visual stimulation (Hogie et al. 2009).

Studies with Swiss-Webster mice found that the locomotor activity of the animals treated with toluene was increased during exposure to toluene concentrations of 0, 2000 or 4000 ml/m^3^ for 30 minutes and a subsequent 30-minute observation period. Dopamine neurotransmission was increased only in the animals exposed to a toluene concentration of 4000 ml/m^3^ (Apawu et al. 2015).

Groups of 16 C57BL/6 and DKO mice (C57BL/6 double knockouts without calcium/calmodulin-stimulated adenylyl cyclases 1 and 8) were exposed to toluene concentrations of 0, 500, 1000, 2000, 6000 or 8000 ml/m^3^ during the light phase of the day cycle (12 noon to 5 p.m.). Both C57BL/6 and DKO mice exhibited increased locomotor activity after exposure to a toluene concentration of 2000 ml/m^3^. At higher concentrations, toluene had a sedating effect on C57BL/6 and DKO mice during the exposure and recovery period (Conti et al. 2012).

RD_50_ values of 12 590, 12 650 and 19 875 mg/m^3^ (3300, 3314 and 5200 ml/m^3^) were determined for mice (EU 2003).

#### 5.1.2 Oral administration

A study administered toluene to 16 Wistar albino rats in gavage doses of 0 or 5200 mg/kg body weight. Blood and liver samples were analysed after 3 hours. Aspartate and alanine aminotransferase activities in the blood were increased with statistical significance. The histopathological examination found slight hepatocyte degeneration and mononuclear cell infiltration in the liver tissues in comparison with the findings in the controls, as well as an increased expression of the apoptosis-mediating proteins Bax and caspase-1 (Ayan et al. 2013).

Toluene (6 ml/kg body weight, equivalent to 5220 mg/kg body weight) was given by gavage to 10 Wistar albino rats. There was also a group of 10 control animals. The experiment was ended 150 minutes after administration. Every 30 minutes, the blood pressure and the heart rate of the animals were monitored for a period of 90 minutes. The mean blood pressure, but not the heart rate, of the animals treated with toluene was decreased with statistical significance in comparison with the values determined in the control animals. The histopathological examination of the heart tissue revealed oedema and thickened areas. The number of apoptotic cells in the hearts of the animals treated with toluene, as determined by TUNEL and caspase-3 assay, was increased with statistical significance in comparison with the values found in the control animals (Tas et al. 2013 a).

#### 5.1.3 Dermal application

There are no new studies available.

#### 5.1.4 Intraperitoneal injection

Sprague Dawley rats (number of animals not specified) were given a toluene dose of 1100 mg/kg body weight by intraperitoneal injection. The number of hydroxyl radicals in the prefrontal cortex was not increased and lipid peroxidation (determined as free malondialdehyde levels) was slightly reduced in the cerebellum (Chalansonnet et al. 2013).

### 5.2 Subacute, subchronic and chronic toxicity

#### 5.2.1 Inhalation

A study in Swiss Webster mice found that exposure to toluene concentrations of 2000 or 4000 ml/m^3^ for 30 minutes a day on 7 days led to a concentration-dependent decrease of 25% to 50% in evoked dopamine release in the nucleus accumbens core and shell in comparison with the levels determined in the control animals, but had no effects on the stimulated release of dopamine in the putamen. An increase in the locomotor activity of the exposed animals was likewise observed (Apawu et al. 2015).

Male C3H/HeN mice (n = 5–10) were exposed to toluene concentrations of 0 or 9 ml/m^3^ for 30 minutes on 3 consecutive days followed by weekly sessions for 3 weeks. The mice were additionally treated either with rat IgG, ovalbumin or anti-CD4 antibodies. The levels of the growth factor BDNF (brain-derived neurotrophic factor) in the bronchoalveolar lavage fluid and of the nerve growth factor (NGF) in plasma were increased with statistical significance. After treatment with anti-CD4 antibodies, these effects were no longer detected in the mice treated with toluene. These findings suggest that after exposure to toluene the CD4^+^ T cells are involved in the production of neurotrophin (Fujimaki et al. 2009).

Toluene impairs chemosensory sensitivity. Female Crlf:OF-1 mice were exposed to toluene concentrations of 0 or 1000 ml/m^3^ for 5 hours a day, on 5 days a week, for 4 weeks. During exposure and the two following weeks, performance in the labyrinth test was found to be decreased with statistical significance. The performance of the mice was not decreased 4 weeks after the end of exposure. The histopathological examination revealed a decrease in the number of cells and in the thickness of the olfactory epithelium during the treatment period. At the end of the recovery period, the thickness of the epithelium was in the normal range, but the density of the cells was lower than before exposure (Jacquot et al. 2006).

Groups of 10 female Crlj:C3H/HeN mice were exposed to toluene concentrations of 0 or 50 ml/m^3^ for 6 hours a day, on 6 days a week for a total of 6 or 12 weeks. The total number of cells and the number of macrophages were increased and the interferon-gamma and substance P concentrations were decreased with statistical significance in the bronchoalveolar lavage fluid after exposure for 6 and 12 weeks. Neutrophin-3 concentrations were increased only after exposure for 12 weeks (Fujimaki et al. 2007).

In a 14-week inhalation study, groups of 10 female and 10 male B6C3F1 mice were exposed to toluene concentrations of 0, 100, 625, 1250, 2500 or 3000 ml/m^3^ for 6.5 hours a day on 5 days a week. In the first 2 weeks, 60% of the male mice and 100% of the female mice in the 3000 ml/m^3^ group and 70% of the female mice in the 2500 ml/m^3^ group died. The body weights in all exposed groups were 7% to 13% lower than those of the controls. In comparison with the values determined in the controls, the relative liver weights were increased with statistical significance after exposure to toluene concentrations of 625 ml/m^3^ and above; at toluene concentrations of 1250 ml/m^3^ and above, the relative lung weights (9% to 15%) were increased with statistical significance, as were the relative kidney weights, but the latter only in female mice. However, the increased lung weights were not accompanied by an increased incidence of histopathological lesions in the lungs, trachea or nose. In the groups of male mice, all animals exposed to a toluene concentration of 2500 and 70% of those exposed to 3000 ml/m^3^ developed centrilobular hypertrophy of the liver (NTP 1990).

Exposure of groups of 10 female and 10 male B6C3F1 mice to toluene concentrations of 0, 600 or 1200 ml/m^3^ (6.5 hours/day, 5 days/week) by inhalation over a period of 15 months did not lead to consistent haematological effects or changes in organ weights. In the animals exposed to a toluene concentration of 1200 ml/m^3^, 40% of the female mice developed minimal hyperplasia of the bronchial epithelium (NTP 1990).

Male F344 rats (6 per concentration and duration) were exposed to toluene concentrations of 0 or 1000 ml/m^3^ for 6 hours a day, for 3 or 7 days. The corticosterone concentrations were increased with statistical significance in the same rats that exhibited decreases in brain GFAP (glial fibrillary acidic protein). These results show that decreases in brain GFAP may be a consequence of a disruption of the hypothalamic–pituitary–adrenal axis and hormonal homeostasis (Little et al. 1998).

Male pigmented DA/HAN rats (5/group) were exposed to concentrations of 0 or 100 ml/m^3^ on 5 days for 3 hours a day. At the end of exposure, the eye position at rest was unsteady, the eye velocity was slower and the eye movements were more irregular in response to visual stimulation; these effects persisted after the end of visual stimulation (Hogie et al. 2009).

Sprague Dawley rats (8/group) were exposed to toluene concentrations of 0 or 1000 ml/m^3^ for 6 hours a day, on 5 days a week, for 10 days. The extracellular malondialdehyde levels were quantified by microdialysis of the prefrontal cortex. Free and total malondialdehyde were determined in different areas of the brain such as the frontal and prefrontal cortex, striatum and cerebellum. The levels of malondialdehyde were not found to be increased after exposure to toluene (Chalansonnet et al. 2013).

Groups of 8 to 24 male Long Evans rats were exposed to toluene concentrations of 0, 1000, 1250, 1500, 1750 or 2000 ml/m^3^ for 6 hours a day, on 5 days a week, for 4 weeks. The audiometric thresholds were determined 24 to 32 hours after exposure. The ears of the animals were examined histopathologically directly after the end of exposure. In all rats treated with toluene, loss of outer hair cells in the organ of Corti was observed in the middle and mid-apical turns of the cochlea. The effect increased with the concentration from the first to the third row of hair cells. At toluene concentrations of 1750 ml/m^3^ and above, the determination of the audiometric thresholds revealed a significant impairment of the hearing ability in the 8 to 24 hertz range. The authors suggest that the absence of any adverse effects on hearing at low concentrations in spite of the evidence of histological changes were due to the inadequate sensitivity of the determination method used (Campo et al. 1997).

A group of 7 Wistar albino rats were treated in a study with toluene concentrations of 0 or 3000 ml/m^3^ for 1 hour a day for 30 days. Pronounced degeneration and decreased PAS (periodic acid–Schiff’s reagent) reactivity were found in the liver cells of the treated animals. Apoptotic activity was increased in the TUNEL test (Tas et al. 2013 b).

Groups of 10 male Wistar rats were exposed to toluene concentrations of 0 or 3000 ml/m^3^ (8 hours/day, 6 days/week) for 12 weeks. The histopathological examination of the lungs found inflammatory effects in the animals exposed to toluene including inflammatory cell infiltration in peribronchial and alveolar lung regions, alveolar septal infiltration, alveolar oedema and exudate, interstitial fibrosis and necrosis (Kanter 2011 b). Severe degenerative changes were observed in the neurons of the frontal cortex, such as shrunken cytoplasm, severely dilated cisternae of the endoplasmic reticulum (ER) and swollen mitochondria with degenerated cristae, the breakdown of the nuclear membrane and disorganization of the chromatin (Kanter 2011 a). Enlarged sinusoids filled with blood were found in the liver. In addition, in some of the hepatocytes a loss of cytoplasm and a hyperchromatic nucleus were observed (Kanter 2012). Mitochondrial degeneration, the dilation of smooth endoplasmic reticulum and enlarged intercellular spaces in both Sertoli and spermatid cells were also detected (Kanter 2011 c). In addition, increased apoptosis was found in all organs in comparison with the levels determined in the controls (Kanter 2011 a, b, c, 2012).

Groups of 6 female and 6 male Sprague Dawley rats were exposed to toluene concentrations of 0 or 40 ml/m^3^ for 16 weeks. Toluene did not cause any changes in locomotor activity, but decreased the rearing activity with statistical significance. Narcosis induced by acute exposure to toluene was observed at lower toluene concentrations in the animals pretreated for 16 weeks than in the control animals. In addition, toluene altered neurotransmission in various areas of the brain (Berenguer et al. 2003, 2004). Concurrent exposure to noise (80 dBA) and a toluene concentration of 40 ml/m^3^ did not increase the severity of the effects induced by toluene (Berenguer et al. 2004).

Inhalation exposure of groups of 10 female and 10 male F344/N rats to toluene concentrations of 0, 100, 625, 1250, 2500 or 3000 ml/m^3^ (6.5 hours/day, 5 days/week) for 15 weeks led to statistically significant increases in the relative liver, kidney and heart weights at toluene concentrations of 1250 ml/m^3^ and above and to a 14% to 25% decrease in body weights at 2500 ml/m^3^ and above. However, histopathological changes in the lungs, trachea, nose and heart were not observed. The number of leukocytes in the blood was found to be reduced in female animals at toluene concentrations of 1250 ml/m^3^ and above. Eight of 10 male rats died in the second week of exposure to a toluene concentration of 3000 ml/m^3^ (NTP 1990).

Inhalation exposure of groups of 10 female and 10 male F344/N rats to toluene concentrations of 0, 600 or 1200 ml/m^3^ (6.5 hours/day, 5 days/week) for 15 months did not induce consistent haematological effects or changes in organ weights. Exposure to toluene increased the incidence and severity of non-neoplastic lesions in the nasal cavity such as degeneration of the olfactory and respiratory epithelium and goblet cell hyperplasia. Additionally, the severity of nephropathy was marginally increased in the females. Toluene-induced neoplasia was not observed (NTP 1990).

#### 5.2.2 Oral administration

Groups of 10 female and 10 male B6C3F1 mice and F344 rats were given gavage doses of toluene of 0, 312, 625, 1250, 2500 or 5000 mg/kg body weight and day on 5 days a week for 13 weeks. All rats in the high dose group died during the first week. The dose of 2500 mg/kg body weight and day was lethal for 1/10 female rats and 8/10 male rats before the end of the study. In comparison with the findings in the controls, the effects observed at toluene doses of 625 mg/kg body weight and day and above were statistically significant and included increased relative liver, kidney and heart weights, reduced body weights, necrosis in the brain and bleeding in the bladder (NTP 1990).

All mice treated with the highest toluene dose of 5000 mg/kg body weight and day died during the first week; 40% of the mice administered a toluene dose of 2500 mg/kg body weight and day and 10% of the female mice given a toluene dose of 1250 mg/kg body weight and day died before the end of the study. The body weights of the male mice in the 2500 mg/kg group were 16% lower than the weights of the controls. The relative liver weights of the mice were increased at toluene doses of 1250 and 2500 mg/kg body weight and day (NTP 1990).

Other studies with gavage administration are summarized in ATSDR (2017).

#### 5.2.3 Dermal application

There are no data available.

### 5.3 Local effects on skin and mucous membranes

#### 5.3.1 Skin

Animal studies demonstrated that toluene induces irritation of the skin in rabbits, mice and guinea pigs. One of these studies was carried out according to EU Test Guideline B.4 (ECHA 2020; EU 2003).

Toluene in concentrations of 10%, 50% or 100% was applied to the ears of female BALB/c mice once a week for 5 weeks. The substance induced only mild swelling of the skin. Histopathological examination revealed the marginal invasion of inflammatory cells only after treatment with 100% toluene (Saito et al. 2011). A single application of 20 μl undiluted toluene to the skin of the ears of Swiss mice increased the ear thickness. The maximum degree of swelling was reached 30 minutes after application. In TRPA1 (transient receptor potential channel ankyrin type-1) knockout mice, the same treatment led to a much weaker reaction in the mouse ear (75% less swelling in comparison with that observed in wild type Swiss mice). In wild type mice, topical pretreatment with a TRPA1 antagonist led to a 50% weaker response in comparison with the effects of treatment with toluene alone. The authors concluded from these findings that the activation of the TRPA1 receptor is involved in the development of the symptoms of irritant-mediated contact dermatitis such as oedema, pain and neurogenic inflammation (Norões et al. 2019).

#### 5.3.2 Eyes

In a study carried out according to OECD Test Guideline 405, the application of 0.1 ml toluene was slightly irritating to the eyes of 3 of 6 rabbits. In view of the mean irritation indices (after 24, 48 and 72 hours) of 1.47 for redness and of 0.39 for swelling, the substance was not labelled as an eye irritant. In another study with 3 female rabbits, 0.1 ml toluene instilled into the conjunctival sac was found to be moderately to severely irritating to the eyes with effects on the cornea; however, the effects were reversible within 21 days (no other details) (ECHA 2020; EU 2003).

### 5.4 Allergenic effects

#### 5.4.1 Sensitizing effects on the skin

The dataset of the European Chemicals Agency (ECHA) includes the negative results obtained by a maximization test carried out according to OECD Test Guideline 406. In this test, the test substance was applied as a 10% formulation in corn oil for intradermal application and in undiluted form for topical application. The challenge treatment was carried out with a 25% and 50% formulation in corn oil. On examination after 24 and 48 hours, a reaction to the 50%, but not to the 25% formulation, was produced by 1 of 200 guinea pigs (ECHA 2020).

#### 5.4.2 Sensitizing effects on the airways

There are no studies available.

### 5.5 Reproductive and developmental toxicity

An extensive monograph of studies that investigated the reproductive toxicity of toluene is included in ATSDR (2017). The most important studies are summarized below.

#### 5.5.1 Fertility

Only a few studies reported effects on the female and male reproductive organs after inhalation of toluene in concentrations of 2000 ml/m^3^ and above. In female rats, the effects included histological changes in the reproductive organs (antral follicles of the ovaries with abundant vacuoles, lytic areas and degenerative mitochondria) (Tap et al. 1996) and in male rats, a reduction in the number, motility and quality of sperm in addition to changes in the weights and histology of the reproductive organs (Kanter 2011 c; Ono et al. 1996, 1999). After exposure to a concentration of 2000 ml/m^3^ for 60 days before mating, the changes in the sperm count and epididymal weights were not accompanied by effects on fertility (Ono et al. 1996). Most of the studies in rats did not detect any effects on mating and fertility after repeated inhalation of 1200 to 2000 ml/m^3^ (HRC 1991; IRDC 1985; Ono et al. 1996; Roberts et al. 2003; Thiel and Chahoud 1997).

No effects on litter parameters were determined in rats and mice in the concentration range from 50 to 12 000 ml/m^3^ (Bowen et al. 2005, 2007, 2009 a, b; Bowen and Hannigan 2013; Courtney et al. 1986; Dalgaard et al. 2001; Hougaard et al. 2003; HRC 1991, 1992; Jones and Balster 1997; Klimisch et al. 1992; Ladefoged et al. 2004; Litton Bionetics Inc 1978; Ono et al. 1995; Roberts et al. 2007; Saillenfait et al. 2007; Thiel and Chahoud 1997) in spite of concurrent maternal toxicity in the form of reduced body weight gains at concentrations as low as 1200 ml/m^3^ (Dalgaard et al. 2001; HRC 1991; Ono et al. 1995; Roberts et al. 2007; Saillenfait et al. 2007; Thiel and Chahoud 1997).

#### 5.5.2 Developmental toxicity

An extensive monograph of studies that investigated developmental toxicity is included in the ATSDR report on toluene (ATSDR 2017).

The studies of developmental toxicity induced by inhalation exposure are shown in Table 1; those with oral administration can be found in Table 2. Table 3 compares the findings of the studies of developmental toxicity and developmental neurotoxicity.

##### 5.5.2.1 Inhalation

###### Prenatal development

The studies reviewed in the supplement published in 1993 (Greim 1996) that are relevant for the present evaluation are briefly described again below.

In a study carried out according to OECD Test Guideline 414, Sprague Dawley rats were exposed whole-body to toluene concentrations of 0 (controls), 250, 750, 1500 or 3000 ml/m^3^ for 6 hours a day from gestation days 6 to 15. The dams were lethargic at concentrations of 750 ml/m^3^ and above; locomotor disorders, hypersensitivity to acoustic stimuli and reduced body weight gains were observed at concentrations of 1500 ml/m^3^ and above. The NOAEC (no observed adverse effect concentration) for maternal toxicity was therefore 250 ml/m^3^. No signs suggestive of prenatal toxicity were found at 250 and 750 ml/m^3^, but embryofoetal development was impaired in a concentration-dependent manner at concentrations of 1500 ml/m^3^ and above to the extent that foetal weights were reduced and the number of foetuses with unossified ribs was increased. Therefore, the NOAEC for developmental toxicity was 750 ml/m^3^. No teratogenicity was observed (HRC 1992; Roberts et al. 1993).

The NOAEC for developmental and maternal toxicity in rabbits exposed by inhalation to concentrations of 0, 30, 100, 300 or 500 ml/m^3^ was determined to be 500 ml/m^3^, the highest concentration tested (Klimisch et al. 1992).

A review released in 1991 by the California Department of Health, United States, summarized and critically evaluated the animal studies and findings in humans that had been published up to that time (Donald et al. 1991). The authors drew particular attention to the questionable quality of several of the animal studies, suggesting that these do not provide an appropriate basis for a risk assessment and for the establishment of limit values. For example, the shift in the foetal rib profile of mice exposed to a concentration of 400 ml/m^3^, which was described as a teratogenic effect by Courtney et al. (1986), was not considered to be an effect of prenatal toxicity, but within the “normal” profile, by Donald et al. (1991). Therefore, 400 ml/m^3^ is accepted as the NOAEC for developmental toxicity for daily exposure of mice for 7 hours.

A review and critical evaluation of the available data were published by the American Conference of Governmental Industrial Hygienists (ACGIH 1991). This panel of experts concluded that the published animal studies demonstrate that the embryo is not more sensitive to the effects of toluene than the dams.

Other studies that were carried out in the low concentration or dose range are summarized below.

In studies of prenatal developmental toxicity, no effects were observed in the foetuses or dams after daily exposure for 6 to 7 hours during gestation, in rats up to a concentration of 750 ml/m^3^ (HRC 1991, 1992; Litton Bionetics Inc 1978; Ono et al. 1995; Roberts et al. 2007; Saillenfait et al. 2007; Thiel and Chahoud 1997), in mice up to a concentration of 400 ml/m^3^ (Donald et al. 1991; Tsukahara et al. 2009; Win-Shwe et al. 2012 a, b; Yamamoto et al. 2009) and in rabbits up to a concentration of 500 ml/m^3^ (highest concentration; Klimisch et al. 1992). At concentrations of 1000 to 3500 ml/m^3^ and above, reduced body weights and delays in growth and in skeletal development were found in the foetuses of rats concurrently with maternal toxicity (reduced body weights) (HRC 1992; Ono et al. 1995; Roberts et al. 2007; Saillenfait et al. 2007; Thiel and Chahoud 1997). An exception to this was presented by a study that did not observe any changes in the body weight gains of dams at a concentration of 1200 ml/m^3^ (Hass et al. 1999). Animal studies did not provide evidence of the induction of embryolethality or abortive effects by toluene in concentrations up to 3500 ml/m^3^. Also, no teratogenicity was determined up to this concentration (ATSDR 2017).

###### Postnatal development

Inhalation studies in rats with exposure to toluene concentrations up to 1200 ml/m^3^ daily for 6 hours during gestation and in some cases during the early period of postnatal development found little evidence of adverse toxic effects on reproduction in adulthood. The female offspring of dams that had been exposed by inhalation to toluene concentrations of 1200 ml/m^3^ for 6 hours a day from gestation days 9 to 21 revealed a statistically significant delay in vaginal opening in comparison with the findings in the controls. However, no additional substance-related effects on mating, fertility and litter parameters were found in the male and female offspring up to the highest concentration tested of 1200 ml/m^3^ (Thiel and Chahoud 1997). No substance-related changes were observed in the male and female offspring of dams that were exposed daily to a toluene concentration of 1200 ml/m^3^ for 6 hours a day from gestation days 7 to 18 (Hass et al. 1999).

**Tab.1:** Inhalation studies of developmental toxicity and developmental neurotoxicity after exposure to low toluene concentrations

Species, strain, number per group	Exposure	Findings	References
rat, Sprague Dawley, 15–19 ♀	**GD 6–20**, 0, 500, 1500 ml/m^3^, whole body, 6 hours/day, purity: 99.7%, examination: GD 21 examined parameters similar to those of OECD Test Guideline 414, developmental toxicity	**500 ml/m^3^: NOAEC developmental toxicity**, **NOAEC maternal toxicity**; **1500 ml/m^3^**: dams: body weight gains ↓; foetuses: body weights ↓; no embryolethality or teratogenicity	Saillenfait et al. 2007
rat, CD Sprague Dawley BR VAF/Plus, 12 ♀	**GD 6–15**, 0, 250, 750, 1500, 3000 ml/m^3^, whole body, 6 hours/day, developmental toxicity	**250 ml/m^3^**: **NOAEC maternal toxicity**; **750 ml/m^3^**: **NOAEC developmental toxicity**; **750 ml/m^3^ and above**: dams: lethargy; **1500 ml/m^3^ and above**: dams: locomotor disorders, hypersensitivity to acoustic stimuli, body weights ↓; foetuses: body weights ↓, number of foetuses with unossified ribs ↑	Greim 1996; HRC 1992; Roberts et al. 1993, 2007
rat, Wistar, 16 ♀	**GD 7–20**, 0, 1500 ml/m^3^, whole body, 6 hours/day, developmental neurotoxicity	**1500 ml/m^3^**: **NOAEC developmental neurotoxicity**, **NOAEC maternal toxicity**, offspring: body weight gains ↓ (PND 7–23), body weights decreased by 8%–9%, not statistically significant (PND 0), cerebellum: caspase-3 activity ↑ (only PND 6); 1 ♀/litter (12 litters): no effects on learning and memory in the open field and Morris water maze tests as adults	Hougaard et al. 2005; Ladefoged et al. 2004
rat, Sprague Dawley, 11 ♀	**GD 7–17**, 0, 600, 2000 ml/m^3^, whole body, 6 hours/day, purity 98%, developmental toxicity and developmental neurotoxicity	**600 ml/m^3^**: **NOAEC developmental toxicity**, dams: haemoglobin levels ↓, body weights ↓; **2000 ml/m^3^**: **NOAEC developmental neurotoxicity**, dams: body weights ↓, feed consumption ↓; foetuses: body weights decreased by 12% at birth, body weights decreased on PND 21: ♂: by 7%, ♀: by 5.5%; no embryolethality, no teratogenicity; developmental neurotoxicity: no effects found in tests of the reflexes, motor activity, learning (Biel water maze) and motor coordination (rotarod)	Ono et al. 1995
rat, Wistar, 18 ♀, 14 ♀ in the control group	**GD 7–PND 18**, 0, 1200 ml/m^3^, whole body, 6 hours/day, developmental toxicity and developmental neurotoxicity	**1200 ml/m^3^**: **NOAEC maternal toxicity**, offspring: body weights decreased by 12% (5.5 g compared with 6.3 g controls) (PND 0), reflex development ↓ (auditory startle response: 24% compared with 57% PND 13); increase in motor activity (open field); ♀: prolonged escape latency in the Morris water maze test (age: 3.5 months)	Hass et al. 1999
rat, Wistar, 23–29 ♀, 38 ♀ in the control group	**GD 9–21**, 0, 300, 600, 1000, 1200 ml/m^3^, whole body, 6 hours/day, developmental toxicity and developmental neurotoxicity	**600 ml/m^3^**: **NOAEC developmental toxicity**, **NOAEC maternal toxicity**; **1000 ml/m^3^**: dams: body weights decreased by 6%, body weight gains decreased by 13.6%; foetuses: body weights decreased by 8% on PND 1 (5.7 g compared with 6.2 g in the controls), reversible from PND 7 onwards; **1200 ml/m^3^**: **NOAEC developmental neurotoxicity**, dams: body weights decreased by 7.5%, body weight gains decreased by 18%; foetuses: body weights decreased by 8% on PND 1 (5.7 g compared with 6.2 g in the controls), reversible from PND 14 onwards; mortality during lactation ↑, delayed incisor eruption and vaginal opening in ♀; no effects on postnatal physical development and in behavioural tests (rotarod, motor activity, learning and memory in the open field and Morris water maze test)	Thiel and Chahoud 1997
rabbit, Himalayan, Chbb:HM, 15–20 ♀	**GD 6–18**, 0, 30, 100, 300, 500 ml/m^3^, whole body, 6 hours/day, purity: 99.9%, developmental toxicity	**500 ml/m^3^**: **NOAEC developmental toxicity**, **NOAEC maternal toxicity**	Greim 1996; Klimisch et al. 1992
mouse, CD-1, 16 ♀, 15 ♀ in the control group	**GD 6–16**, 0, 200, 400 ml/m^3^, whole body, 7 hours/day, developmental toxicity	**200 ml/m^3^**: foetuses: increased number of litters with dilated renal pelvises, not found at higher concentrations; **400 ml/m^3^**: **NOAEC developmental toxicity**, shift in foetal rib profile	Courtney et al. 1986; Donald et al. 1991; Greim 1996
mouse, CD-1, 13 ♀	**GD 12–17**, 0, 200, 400, 2000 ml/m^3^, whole body, 3×1 hour per day, developmental toxicity and developmental neurotoxicity	**400 ml/m^3^**: **NOAEC developmental neurotoxicity/ developmental toxicity**; **2000 ml/m^3^**: **NOAEC maternal toxicity**; offspring: body weight per foetus decreased on PND 0 (determined by weighing individual foetuses of the litter; however, body weights in the litter no different from those of the controls); body weight gains ↓ (PND 1–20), physical development examined: performance in behavioural tests ↓ (righting reflex, grip strength, inverted screen)	Jones and Balster 1997

GD: gestation day; NOAEC: no observed adverse effect concentration; PND: postnatal day

##### 5.5.2.2 Oral administration

The studies of developmental toxicity after oral administration are shown in Table 2 and 3.

Screening studies carried out according to Chernoff and Kavlock in mice given gavage doses of toluene of 1800 mg/kg body weight and day from gestation days 8 to 12 (Seidenberg et al. 1986) or of 2350 mg/kg body weight and day from gestation days 7 to 14 (NIOSH 1983) did not reveal any effects on litter parameters or visceral malformations.

In a study with gavage administration in dams from gestation days 16 to 19, rat foetuses were found to have dilated renal pelvises at 1250 mg/kg body weight and day (Warner et al. 2008). The NOAEL (no observed adverse effect level) for developmental toxicity was 750 mg/kg body weight and day (see Table 2).

**Tab.2:** Studies of developmental toxicity and developmental neurotoxicity after oral administration of toluene

Species, strain, number per group	Exposure	Findings	References
rat, Sprague Dawley, 5 ♀	**GD 16–19**, 0, 250, 750, 1250 mg/kg body weight and day, gavage, developmental toxicity	**750 mg/kg body weight**: **NOAEL developmental toxicity,** **NOAEL maternal toxicity**; **1250 mg/kg body weight**: dams: kidneys: swollen tubules, adhesion of tissue in Bowman’s capsule ↑, foetuses: incidence of dilated renal pelvises ↑	Warner et al. 2008
mouse, CD-1, 10 ♀	**GD 7–14**, 0, 2350 mg/kg body weight and day, gavage, screening test according to Chernoff and Kavlock	**2350 mg/kg body weight**: **NOAEL embryotoxicity and visceral malformations, NOAEL maternal toxicity**, dose-finding study: at 5890 mg/kg body weight and day and above, 8 days: lethal to female mice	NIOSH 1983
mouse, ICR/SIM, 30 ♀	**GD 8–12**, 0, 1800 mg/kg body weight and day, gavage, screening test according to Chernoff and Kavlock	**1800 mg/kg body weight**: **NOAEL embryotoxicity and visceral malformations, NOAEL maternal toxicity**	Seidenberg et al. 1986
mouse, Nya:NYLAR, 12 ♀	**GD 0–21 and PND 0–55**, 0, 16, 80, 400 mg/l in the drinking water (0, 2.9, 14,4, 72 mg/kg body weight and day), postnatal developmental toxicity/developmental neurotoxicity	**14.4 mg/kg body weight**: **NOAEL developmental neurotoxicity**; **72 mg/kg body weight**: **NOAEL for postnatal development**, offspring: reduced habituation in a 20-minute open field activity, no changes in postnatal physical development (eye opening, pinna detachment), righting reflex or startle response; Note: reduced habituation in the open field test is not a reliable end point, the evaluation was not blind; no dose–response relationship in the rotarod test; study questionable	Kostas and Hotchin 1981

GD: gestation day; NOAEL: no observed adverse effect level; PND: postnatal day

##### 5.5.2.3 Developmental neurotoxicity

The studies that investigated developmental neurotoxicity are shown in Table 1, 2 and 3.

###### Inhalation

In rats, the findings in the offspring of dams that were exposed to toluene concentrations of 600 or 2000 ml/m^3^ for 6 hours a day from gestation days 7 to 17 did not differ with statistical significance from those determined in the control animals with regard to the following parameters: postnatal viability or physical development up to postnatal day 21, reflex tests from postnatal days 6 to 10, motor activity during postnatal week 4, balancing on a rotating cylinder (rotarod) in postnatal week 7 and learning in the Biel water maze during postnatal week 6 (Ono et al. 1995). A NOAEC for developmental neurotoxicity of 2000 ml/m^3^ was derived from these findings; this was the highest concentration tested.

In a study with rats, the results of behavioural tests carried out with the offspring of dams that were exposed to toluene concentrations of 300, 600, 1000 or 1200 ml/m^3^ for 6 hours a day from gestation days 9 to 21 did not reveal any consistent, concentration-dependent deficits with respect to the reflexes on postnatal day 3, balancing on a rotating cylinder (rotarod) on postnatal day 18, motor activity from postnatal days 31 to 34 and in learning and memory tests (open field and Morris water maze test) from postnatal days 70 to 81 (Thiel and Chahoud 1997). A NOAEC for developmental neurotoxicity of 1200 ml/m^3^ was derived; this was the highest concentration tested.

In studies with rats, no changes were observed in the Morris water maze test and in the open field test with the female offspring of dams exposed to toluene concentrations of 1500 ml/m^3^ for 6 hours a day from gestation days 7 to 20 (Hougaard et al. 2005; Ladefoged et al. 2004).

The exposure of female rats to a toluene concentration of 1200 ml/m^3^ for 6 hours a day from gestation day 7 to postnatal day 18 led to a statistically significant decrease in body weights from birth to postnatal day 10, a delay in reflex development between postnatal days 2 and 13 and an increase in motor activity in the open field test on postnatal day 28 in male and female offspring in comparison with the findings in the control animals. A statistically significant increase in latency to discover the hidden platform in the Morris water maze test after relocation of the platform was observed in the female offspring at the age of 13 weeks only on a per offspring basis, not on a per litter basis. No statistically significant effects were observed in the rotarod test, in the listening test at 4 months of age and in physical development (eye opening, etc.) (Hass et al. 1999).

In mice, the offspring of dams that were exposed to a toluene concentration of 2000 ml/m^3^ for 60 minutes 3 times a day from gestation days 12 to 17 had lower body weights between postnatal days 2 and 8 in comparison with the body weights determined in the control animals. Other findings were a statistically significant increase in righting reflex latency on postnatal days 1, 5 and 6, reduced grip strength of the forelimbs from postnatal days 5 to 7 and 9 to 11 as well as increased latency in the inverted screen test from postnatal days 14 to 17. Tests carried out according to the same protocol did not reveal any statistically significant effects relating to these end points after exposure to toluene concentrations of 200 or 400 ml/m^3^. No differences were determined in the achievement of developmental milestones such as incisor eruption and eye opening (Jones and Balster 1997). A NOAEC for developmental neurotoxicity of 400 ml/m^3^ was derived from this study.

###### Oral administration

The behavioural development of mice was investigated after exposure to toluene concentrations of 0, 16, 80, 400 mg/l in the drinking water (equivalent to toluene doses of 0, 2.9, 14.4, 72 mg/kg body weight and day) from gestation day 0 to postnatal day 55. In the high dose group with exposure to 72 mg/kg body weight and day, habituation was found to be significantly reduced during a 20-minute open field activity. The authors derived a NOAEL and LOAEL (lowest observed adverse effect level) of 14.4 and 72 mg/kg body weight and day, respectively, for this developmental toxicity test based on the delayed habituation to the surrounding environment in the open field test. No effects were noticeable in the open field test after intraperitoneal administration of an acute dose that was equivalent to the average toluene dose given in the drinking water during the fifth week of treatment (Kostas and Hotchin 1981). As noted in Table 2, delayed habituation in the open field test is not a reliable end point and was not observed after intraperitoneal administration. Furthermore, the evaluation was not blind and no dose–response relationship was observed in the rotarod test. Overall, the findings of this study appear questionable.

###### Studies in animals as models for solvent abuse

A series of studies was carried out to investigate solvent abuse during pregnancy using the rat model. The objective was to determine whether repeated short-term exposure to very high concentrations during gestation would induce developmental toxicity. After exposure to toluene concentrations in the range from 8000 to 16 000 ml/m^3^ twice a day for 15 to 30 minutes from gestation days 6 to 20, maternal toxicity in the form of reduced body weight gains and foetal toxicity in the form of decreases in body weights, crown-to-rump length and postnatal growth were observed. Also, the placental weights were reduced. The number of litters with malformations, runting and neonatal death was increased and an impairment of motor activity, learning and memory performance and a change in reward behaviour with increased impulsivity were observed in the offspring (Bowen et al. 2005, 2007, 2009 a, b; Bowen and Hannigan 2013; Callan et al. 2017; Jarosz et al. 2008). The physical postnatal development remained unaffected (Bowen et al. 2005; Bowen and Hannigan 2013).

###### Summary of developmental neurotoxicity

Only a few behavioural tests (righting reflex, grip strength, inverted screen) revealed deficits in the offspring of mice exposed by inhalation to a concentration of 2000 ml/m^3^ 3 times a day for 60 minutes from gestation days 12 to 17, but none in those exposed to 400 ml/m^3^ (Jones and Balster 1997). No effects were found in behavioural tests carried out with the offspring of rats that were exposed to concentrations up to 2000 ml/m^3^ for 6 hours a day during gestation (Hougaard et al. 2005; Ladefoged et al. 2004; Ono et al. 1995; Thiel and Chahoud 1997). An impairment of motor activity, deficits in learning and memory, an increasing number of malformations and foetal deaths were observed in rats exposed during gestation to high concentrations chosen to replicate solvent abuse scenarios (8000 to 16 000 ml/m^3^, 15 to 30 min a day) (Bowen et al. 2005, 2009 b; Bowen and Hannigan 2013; Callan et al. 2017).

##### 5.5.2.4 PBPK models

The ATSDR report describes several PBPK models (ATSDR 2017). Two studies carried out by the same research group analysed blood concentrations in humans and rats after exposure by inhalation. A comparison of the values determined in rats and humans after exposure to the same toluene concentration of 100 ml/m^3^ for 3 to 4 hours revealed that blood toluene levels were somewhat higher in rats (2–3 mg/l in rats, about 1 mg/l in humans) (Benignus et al. 2006; Kenyon et al. 2008). Wistar rats were found to have blood levels of 2 mg/l after exposure by inhalation to 125 ml/m^3^ for 4 hours (Kishi et al. 1988).

Therefore, at about the same level of exposure, the concentration found in the blood of rats was somewhat higher than that found in humans. Therefore, the evidence does not indicate a marked difference.

**Tab.3:** Comparison of developmental toxicity and developmental neurotoxicity in rats and mice

Developmental toxicity	Developmental neurotoxicity	Comments	References
**mouse**			
**400 ml/m^3^: NOAEC** 2000 ml/m^3^: body weight gains ↓ (PND 1–20)	**400 ml/m^3^: NOAEC** 2000 ml/m^3^: performance in behavioural tests ↓ (righting reflex, grip strength, inverted screen)	large margin between NOAEC and LOAEC; therefore, a higher NOAEC possible; determination of average body weights of offspring questionable: only 1 female and 1 male per litter weighed, no clear effects on foetal weights, foetuses lighter, but the first effect offset by the larger litter sizes	Jones and Balster 1997
**72 mg/kg body weight and day: NOAEL postnatal development toxicity** (highest dose; developmental milestones)	**14.4 mg/kg body weight and day: NOAEL** 72 mg/kg body weight and day: offspring: reduced habituation during a 20-minute open field activity	reduced habituation in the open field test is not a reliable end point, the evaluation was not blind, no dose–response relationship in the rotarod test, study questionable	Kostas and Hotchin 1981
**rat**			
1500 ml/m^3^: body weight gains ↓ (PND 7–23), body weights decreased by 8%–9%, not statistically significant (PND 0)	**1500 ml/m^3^: NOAEC (only 1 concentration)**		Hougaard et al. 2005; Ladefoged et al. 2004
**600 ml/m^3^: NOAEC** 2000 ml/m^3^: body weights decreased by 12% at birth	**2000 ml/m^3^: NOAEC (highest concentration)**		Ono et al. 1995
1200 ml/m^3^: body weights decreased by 12% (5.5 g compared with 6.3 g controls) (PND 0)	1200 ml/m^3^: reflex development ↓ (auditory startle response: 24% compared with 57% PND 13)	reviewed by Thiel and Chahoud (1997): no confirmation of the effects on developmental neurotoxicity by Hass et al. (1999)	Hass et al. 1999
**600 ml/m^3^: NOAEC** 1000 ml/m^3^: body weights decreased by 8% (5.7 g compared with 6.2 g controls) (PND 1) 1200 ml/m^3^: body weights ↓; developmental delays with respect to incisor eruption, vaginal opening	**1200 ml/m^3^: NOAEC (highest concentration),** no effects in behavioural tests up to 1200 ml/m^3^	extensive and well-performed study with a high confidence level	Thiel and Chahoud 1997

### 5.6 Genotoxicity

Several of the studies were described in detail in the documentation from 1986 (Greim 1996), but have been included also in this addendum for comparative purposes. The studies in vitro are shown in Table 4, the studies in vivo in Table 5.

#### 5.6.1 In vitro

Toluene did not induce differential killing, SOS response or prophage induction in bacteria (McCarroll et al. 1981 a, b; Nakamura et al. 1987; Rossman et al. 1991).

In gene mutation tests in Salmonella typhimurium TA98, TA100, TA1535, TA1537, TA1538, UTH8413 and UTH8414, negative results were obtained for toluene concentrations up to 5000_ µ_g/plate (LC_50_: about 4350 µg/plate) both with and without the addition of a metabolic activation system (rat, hamster) (Bos et al. 1981; Connor et al. 1985; Haworth et al. 1983; Litton Bionetics Inc 1983; Nestmann et al. 1980; NTP 1990; Spanggord et al. 1982). As toluene is a volatile substance, liquid preincubation tests (Haworth et al. 1983; Litton Bionetics Inc 1983; NTP 1990) are particularly relevant for the evaluation. However, the findings of the study carried out by Litton Bionetics Inc. (1983) varied extensively and therefore cannot be used for the evaluation.

In a suspension test in Saccharomyces cerevisiae, toluene did not lead to mitotic gene conversions up to the LC_50_ (Litton Bionetics Inc 1983). As the positive control induced fewer revertants than the solvent control in 1 of 2 experiments with a metabolic activation system, this test is regarded as invalid and has not been included in the evaluation.

DNA strand breaks were induced by toluene in human HL-60 cells in the comet assay. The concentrations tested resulted in 20% (105 µg/ml) and 50% (253 µg/ml) cytotoxicity. The number of apoptotic cells, determined by flow cytometry, and the expression of apoptosis-inducing proteins were increased after treatment with toluene in comparison with the findings determined in the controls (Sarma et al. 2011).

Treatment of rat hepatocytes with toluene led to a statistically significant increase in DNA single-strand breaks (alkaline elution) at concentrations of 2.76 µg/ml and above (Sina et al. 1983). The glutamate-oxaloacetate transaminase test used to determine cytotoxicity does not appear to have yielded reliable results; therefore, valid conclusions relating to the cytotoxicity of the tested concentrations cannot be drawn (see Table 4). For this reason, the results are not included in the evaluation.

The 100-fold concentration of 276 µg/ml induced neither DNA strand breaks (nick translation) nor subsequent DNA repair in human fibroblasts (Snyder and Matheson 1985).

Toluene did not induce SCE in CHO cells up to markedly cytotoxic concentrations both with and without the addition of a metabolic activation system (NTP 1990) or in primary human lymphocytes in concentrations from 4.6 to 92 µg/ml (Richer et al. 1993) and 15.2 to 1520 µg/ml (Gerner-Smidt and Friedrich 1978). Toluene did not have any effects on the frequency of chromosomal aberrations in CHO cells (Richer et al. 1993) or in primary human lymphocytes in the same concentration range, but did inhibit cell growth (Gerner-Smidt and Friedrich 1978).

In a micronucleus test with cytochalasin B in various human cell lines, the number of micronuclei induced by toluene increased with the metabolic competency of the cells. The frequency of micronuclei was increased with statistical significance in transfected human lymphoblasts with higher levels of CYP1A1 activity and stable expression of cDNA encoding different xenobiotic enzymes (CYP2E1 in 2hE1 cells; CYP1A2, CYP2A6, CYP3A4, CYP2E1 and microsomal epoxide hydrolase in MCL-5 cells) in comparison with the frequency determined in the parental cell line AHH-1, which has only low native CYP1A1 activity. All cell lines formed more kinetochore-negative and centromere-negative micronuclei than kinetochore-positive and centromere-positive micronuclei; in comparison with the findings in the controls, the latter were increased with statistical significance in the cell lines only at levels of cytotoxicity above 62% (5 mM). The cytotoxicity, determined as the fraction of binuclear cells, increased in a concentration-dependent manner from 3% to 11% at 0.01 mM and from 62% to 75% at 5 mM (AHH-1: 62%; h2E1: 75%; MCL-5: 66%) in comparison with the levels found in the controls (Doherty et al. 1996).

The frequency of micronuclei was not increased in human blood lymphocytes from 4 donors after treatment with toluene concentrations of 9.2 to 184 µg/ml without the addition of a metabolic activation system (Zarani et al. 1999). The results from the assay that used a metabolic activation system are considered invalid, as the lymphocytes of 1 donor were evaluated only once.

In a study with and without metabolic activation, positive results were obtained for toluene in a TK^+/–^ mutation test in L5178Y cells only at markedly cytotoxic concentrations; no differentiation into small and large colonies was made. Statistical significance was reached at toluene concentrations of 200 µg/ml and above, which at the same time decreased viability to 30% to 40%. Also, the authors suggest that a toluene and water emulsion may have formed, which would have changed the concentrations tested (McGregor et al. 1988; NTP 1990).

None of the other in vitro studies that tested concentrations in this range and higher in cell culture media reported the formation of an emulsion and this point was generally not discussed.

Another TK^+/–^ mutation test in the same cell type yielded negative results up to markedly cytotoxic concentrations both with and without the addition of a metabolic activation system. Viability was below 10% at the high concentration of 260 µg/ml (Litton Bionetics Inc 1983).

**Tab.4:** In vitro genotoxicity of toluene

End point	Test system	Concentration	Effective concentration	Cytotoxicity	Results		Comments	References
					**–m. a.**	**+m. a.**		
prophage induction	Escherichia coli WP2s (λ) (microtiter plate)	experiment 1: 0, 0.78–100 µg/well; experiment 2: highest concentration tested limited by solubility or cytotoxicity, no other details	–	no data	–	–		Rossman et al. 1991
prophage induction and UMU test	Salmonella typhimurium TA1535/pSK 1002 *umu*	≤ 100 µg/ml	–	no data	–	–		Nakamura et al. 1987
differential killing (rec assay)	Bacillus subtilis *rec^+/–^*	≤ 20 000 µg/well	–	20 000 µg/well	–	–		McCarroll et al. 1981 a
differential killing (rec assay)	Escherichia coli WP2, WP2 *uvrA^–^*, WP67 *uvrA^–^ pol^–^*, CM611 *uvrA^–^ lexA^–^*, WP100 *uvrA^–^ recA^–^*, W3110 *polA^+^, p3478 polA^–^*	≤ 60 000 µg/well	–	60 000 µg/well	–	–		McCarroll et al. 1981 b
gene mutation (liquid preincubation test)	Salmonella typhimurium TA98, TA100, TA1535, TA1537	0, 10–1000 µg/plate	–	1000 µg/plate	–	–	S9 mix: rat, hamster	Haworth et al. 1983; NTP 1990
gene mutation (plate incorporation)	Salmonella typhimurium TA98, TA100, TA1535, TA1537, TA1538	no data	–	4300 µg/plate	–	–		Nestmann et al. 1980
	Salmonella typhimurium TA98, TA100, TA1535, TA1537, TA1538	0, 100–2000 µg/plate	–	0%–20% at 2000 µg/plate	–	–	S9 mix: rat, treated and untreated with Aroclor	Bos et al. 1981
	Salmonella typhimurium TA98, TA100, TA1535, TA1537, TA1538	0, 10–5000 µg/plate	–	no data	–	–		Spanggord et al. 1982
	Salmonella typhimurium TA98, TA100, TA1535, TA1537, TA1538, UTH8413, UTH8414	0, 50–2000 µg/plate	–	2000 µg/plate	–	–	purity > 99%	Connor et al. 1985
gene mutation (plate incorporation and liquid preincubation test)	Salmonella typhimurium TA98, TA100, TA1535, TA1537, TA1538	0, 0.87–4350 µg (0.001–5 µl)/plate	–	C_max_ = LD_50_	–^a)^	–^a)^		Litton Bionetics Inc 1983
mitotic gene conversion	Saccharomyces cerevisiae D4	0%, 0.1375%–1.1% (v/v)	–	C_max_ = LD_50_	–	(–)^b)^		Litton Bionetics Inc 1983
DNA strand breaks (comet assay)	human acute myeloid leukaemia cells (HL-60)	0, 1.14, 2.74 mM (105, 253 µg/ml)	≥ 105 µg/ml	105 µg/ml: IC_20_; 253 µg/ml: IC_50_	+	n. t.		Sarma et al. 2011
DNA strand breaks (alkaline elution)	rat hepatocytes	0, 0.03, 0.3, 3 mM (2.76, 27.6, 276 µg/ml)	≥ 2.76 µg/ml	2.76, 27.6 µg/ml: about 85%; 276 µg/ml: about 50%	+^c)^	n. t.	cytotoxicity by glutamate oxaloacetate transaminase test	Sina et al. 1983
DNA strand breaks (nick translation)	human fibroblasts	0, 3 mM (276 µg/ml)	–	no data	–	n. t.		Snyder and Matheson 1985
sister chromatid exchange	CHO	0, 16–5000 µg/ml	–	–m. A.: ≥ 400 µg/ml; +m. A.: ≥ 500 µg/ml	–	–		NTP 1990
	primary human lymphocytes (blood)	0, 15.2, 152, 1520 µg/ml	–	≥ 152 µg/ml: ≥ 5% inhibition of cell growth; no other details	–	n. t.		Gerner-Smidt and Friedrich 1978
	primary human lymphocytes (blood)	0, 0.05, 0.5, 1, 2.5 mM (4.6, 46, 92, 230 µg/ml)	–	4.6 µg/ml: about 10%; 230 µg/ml: 100%	–	n. t.		Richer et al. 1993
chromosomal aberrations	CHO	0, 50–1600 µg/ml	–	–	–	–		NTP 1990
	primary human lymphocytes (blood)	0, 15.2, 152, 1520 µg/ml	–	≥ 152 µg/ml: ≥ 5% inhibition of cell growth; no other details	–	n. t.		Gerner-Smidt and Friedrich 1978
micronuclei (kinetochore and centromere labelling)	human lymphoblasts (AHH-1)	0, 0.01, 0.1, 1, 2, 5 mM (0.92, 9.2, 92, 184, 460 µg/ml)	K–ve/ K+ve: 5 mM	5 mM: 62%	+	n. t.	positive results with statistical significance only with marked cytotoxicity	Doherty et al. 1996
	human lymphoblasts (MCL-5)	0, 0.01, 0.1, 1, 2, 5 mM (0.92, 9.2, 92, 184, 460 µg/ml)	K–ve: 1 mM; K+ve: 5 mM	1 mM: 32%; 5 mM: 66%	+	n. t.		
	human lymphoblasts (h2E1)	0, 0.01, 0.1, 1, 2, 5 mM (0.92, 9.2, 92, 184, 460 µg/ml)	K–ve: 1 mM; K+ve: 5 mM	1 mM: 36%; 5 mM: 75%	+	n. t.		
micronuclei	human lymphocytes	0, 0.1, 0.5, 1, 2 mM (9.2, 46, 92, 184 µg/ml)	–	no data	–	(–)^d)^		Zarani et al. 1999
gene mutation (TK^+/–^)	mouse lymphoma cells (L5178Y)	–m. A.: 0, 31.25–500 µg/ml; +m. A.: 0, 6.25–250 µg/ml	–/+m. A.: ≥ 200 µg/ml	200 µg/ml: ≥ 60%–70%; 275 µg/ml and above: lethal	(+)^e)^	(+)^e)^		McGregor et al. 1988; NTP 1990
	mouse lymphoma cells (L5178Y)	0, 0.05, 0.1, 0.15, 0.2, 0.3 µl/ml (44 to 260 µg/ml)	–	0.15, 0.2 µl/ml: about 50%; 0.3 µl/ml: > 90%	–	–		Litton Bionetics Inc 1983

a) wide range of variation in the liquid preincubation test

b) The positive controls yielded fewer revertants than the solvent controls in 1 of 2 tests with S9.

c) The values determined by the cytotoxicity tests suggest that the system was invalid. Therefore, no conclusions can be drawn on cell viability based on statistically significant results.

d) not meaningful, as only one experiment was carried out with a sample with S9

e) Positive results only at markedly cytotoxic concentrations. Also, the authors were not able to exclude the possibility that an emulsion formed; therefore, no conclusions can be drawn about the concentrations actually attained. Solubility of toluene in water is about 0.52 mg/ml

K–ve/K+ve: kinetochore-negative and centromere-negative and kinetochore-positive and centromere-positive micronuclei; m. A.: metabolic activation, if not specified further, S9 mix from rat liver after administration of polychlorinated biphenyls

#### 5.6.2 In vivo

Toluene did not induce X-chromosomal recessive lethal mutations or translocations at concentrations up to the LD_50_. However, the substance did induce numerical chromosomal aberrations in male Drosophila melanogaster at 66% of the LD_50_ and above (Rodriguez Arnaiz and Villalobos Pietrini 1985 a, b). In another study that was published only as an abstract, toluene likewise yielded negative results in the sex-linked recessive lethal (SLRL) test (Donner et al. 1981).

A large number of studies did not detect chromosomal aberrations or micronuclei in the bone marrow of rats or mice treated by oral, inhalation and intraperitoneal routes of administration. In many cases, the studies do not include data for cytotoxicity in the bone marrow; however, on the basis of toxicokinetics studies, the bone marrow is assumed to be reached because toluene tends to accumulate not only in the fatty tissue and in the brain, but also in the bone marrow (ATSDR 2017).

In mice (n = 3) that were exposed by inhalation to a toluene concentration of 250 ml/m^3^ for up to 8 weeks, DNA strand breaks were not induced in the liver, blood or bone marrow in comparison with the findings in the controls. Pooled samples from groups of 3 animals were analysed together. Toluene did not cause anaemia and did not have an effect on the number of lymphocytes and granulocytes in the blood. After 8 weeks, but not after 4, the number of erythroid burst forming units (BFU-E) was significantly reduced in the bone marrow of animals exposed to a toluene concentration of 500 ml/m^3^ (Plappert et al. 1994).

Positive results obtained in the TUNEL assay revealed DNA strand breaks in the brain, heart, liver, lungs and testes of male rats treated with toluene in vivo. However, the findings of the histopathological examinations, or the presence of markers specific to apoptosis (caspase-3), indicate that the DNA strand breaks result from apoptotic mechanisms and were not induced by genotoxicity (Kanter 2011 a, b, c, 2012; Tas et al. 2013 a, b).

A study that was published only as a conference abstract did not report any effects on the incidence of chromosomal aberrations in the bone marrow of male Wistar rats after inhalation exposure to a toluene concentration of 300 ml/m^3^ for 15 weeks. A test for SCE yielded positive results after 11 and 13 weeks, but not after 15 weeks (Donner et al. 1981).

Another study that was published only as an abstract found that 10 oral doses at levels up to 20% of the LD_50_ did not significantly increase the incidence of chromosomal aberrations in male SHR mice (Feldt et al. 1985).

Chromosomal aberrations were not induced in the bone marrow of rats intraperitoneally administered single (evaluated 6, 24 or 48 hours post-administration) or multiple (1/day; for 5 days) doses of toluene at levels of 22 to 215 mg/kg body weight. Cytotoxicity, expressed as a reduction in the mitotic index, was induced at toluene doses of 71 mg/kg body weight and above, both after administration of single and of multiple doses of toluene (Litton Bionetics Inc 1983). Therefore, it is assumed that toluene or its metabolites reach the bone marrow.

A test for chromosomal aberrations in the bone marrow yielded negative results in mice given 2 oral doses of toluene of 1720 mg/kg body weight within a 30-hour period. Cytotoxic effects were not evaluated (Gad-El Karim et al. 1984).

In a number of earlier studies carried out in the former Soviet Union, chromosomal damage was induced in the bone marrow and blood of rats that were either given daily subcutaneous doses of toluene of 800 to 1000 mg/kg body weight for 5 to 12 days or were exposed daily by inhalation to a toluene concentration of 162 ml/m^3^ for 16 weeks (Dobrokhotov 1972; Dobrokhotov and Enikeev 1977; Lyapkalo 1973). In another study in rats, inhalation exposure to 1.4 ml/m^3^ for 16 weeks did not have statistically significant effects on chromosomal aberrations (Aristov et al. 1981). Cytotoxicity was not evaluated in any of the studies and the types of damage that were analysed are not considered chromosomal aberrations by today’s standards. The studies are therefore not relevant for the evaluation.

In a chromosomal aberration test in the bone marrow of rats that had been given 2 intraperitoneal injections of toluene at dose levels of up to 435 mg/kg body weight within a 30-hour period, positive findings that reached statistical significance were only induced by toluene concurrently with cytotoxicity (Roh et al. 1987). However, as specific lesions (chromosomal breaks) and unspecific lesions (gaps) were reported in sum, the test is not relevant for the evaluation.

A concentration-dependent increase in micronuclei in the polychromatic erythrocytes was induced by toluene in the bone marrow of male NMRI and B6C3F1 mice given 2 intraperitoneal injections of toluene at an interval of 24 hours at dose levels of 0.12, 0.25, 0.37 or 0.5 ml/kg body weight (104, 218, 322, 435 mg/kg body weight). The incidence was determined 30 hours after the first injection. In NMRI mice, the findings differed significantly from those determined in the controls at doses of 0.25 ml/kg body weight and above and in B6C3F1 mice at doses of 0.12 ml/kg body weight and above. In both strains, the maximum number of micronuclei were found at the second highest dose. The preliminary studies determined LD_50_ values of 1.64 ± 0.09 ml/kg body weight for NMRI mice and of 1.88 ± 0.07 ml/kg body weight for B6C3F1 mice (Mohtashamipur et al. 1985).

In another micronucleus test, the frequency of micronuclei was found to be increased with statistical significance in male NRMI mice treated 30 hours prior to analysis either with a single dose of toluene of 0.5 ml/kg body weight (435 mg/kg body weight) or with two doses of either 0.37 (322 mg/kg body weight) or 0.5 ml (435 mg/kg body weight)/kg body weight at an interval of 24 hours. The findings after treatment with two doses of 0.25 ml/kg body weight (218 mg/kg body weight) did not differ with statistical significance from the findings in the controls. Again, the determinations were carried out 30 hours after the first injection. The frequency of micronuclei was increased if the CYP modulators Aroclor 1254, phenobarbital, 3-methylcholanthrene, alpha-naphthoflavone or metyrapone were administered by injection 24 hours prior to the administration of toluene. The simultaneous administration of toluene and alpha-naphthoflavone or metyrapone resulted in micronuclei levels similar to those found after treatment with toluene alone (Mohtashamipur et al. 1987). Cytotoxic effects of toluene have not been investigated in this study.

In rats, toluene administered intraperitoneally in 2 doses of 218 mg/kg body weight led to a statistically significant increase in the number of micronuclei in the bone marrow in comparison with the number found in the controls; however, also the ratio of polychromatic to normochromatic erythrocytes was decreased with statistical significance at this dose and above (Roh et al. 1987).

A single or 2 oral doses of toluene of up to 1720 mg/kg body weight did not induce micronuclei in the bone marrow of male and female CD1 mice. There are no data for cytotoxicity (Gad-El Karim et al. 1984, 1986).

In 2 studies with male CD-1 mice (10–12 per concentration), inhalation exposure to a toluene concentration of 100 ml/m^3^ (6 hours/day, 8 days, either consecutive or over a period of 15 days) did not lead to an increased formation of micronuclei in the blood or bone marrow. Benzene was used as the positive control; the findings confirmed that the test system was functional. The effects on the activities of various enzymes (CYP2E1, EH, GST, NQO1; no data provided) induced by treatment with toluene were inconsistent. Simultaneous depletion of glutathione (GSH) by intraperitoneal administration of buthionine sulfoximine did not affect micronucleus levels in the blood. None of the animals exhibited clinical effects induced by toluene. The ratio of polychromatic to normochromatic erythrocytes remained unchanged in comparison with the ratio determined in the negative controls (Bird et al. 2010; Wetmore et al. 2008).

As published in a conference abstract, oral doses of toluene (0.001–0.2 × LD_50_) given for 5 weeks did not induce dominant lethal mutations in male SHR mice (Feldt et al. 1985).

In another dominant lethal test, subchronic exposure of male CD-1 mice to toluene (100 or 400 ml/m^3^, 6 hours/day, 5 days/week, for 8 weeks) likewise did not lead to the induction of dominant lethal mutations or changes in other parameters (total implants, dead or live implants per pregnant mouse). The positive control confirmed that the test system was functional (Litton Bionetics Inc 1983).

**Tab.5:** In vivo studies of genotoxicity induced by toluene

Test system		Dose/Concentration	Results	Comments	References
gene mutation, germ cells (SLRL)	Drosophila melanogaster (white) ♂	0%, 0.05%, 0.1% in the feed, 24 hours	–	no data for cytotoxicity; > 3000 X chromosomes examined	Donner et al. 1981
gene mutation, germ cells (SLRL)	Drosophila melanogaster (“Oster”)	0%, 0.1%, 0.25%, 0.5%, 0.75%, 1%, 1.25%, 1.5% in the feed, no other data	–	no data for cytotoxicity; C_max_ = LD_50_: 1.5%	Rodriguez Arnaiz and Villalobos Pietrini 1985 b
aneuploidy (non-disjunction or loss of sex chromosomes)	Drosophila melanogaster (“Oster”), ♀, ♂	0%, 0.1%, 0.25%, 0.5%, 0.75%, 1.0% 1.25%, 1.5% in the feed, no other data	♀: – ♂: +	♂: statistical significance at ≥ 1.0%; no data for cytotoxicity; C_max_ = LD_50_: 1.5%	Rodriguez Arnaiz and Villalobos Pietrini 1985 a
DNA strand breaks (comet assay)	mouse, B6D2F1, groups of 3 ♀, blood, bone marrow, liver	0, 250 ml/m^3^, inhalation, 6 hours/day, 5 days/week, 2, 8 weeks	–	cytotoxicity in the bone marrow after 8 weeks; samples from 3 animals analysed together	Plappert et al. 1994
sister chromatid exchange, bone marrow	rat, Wistar, ♂, no other data	0, 300 ml/m^3^, inhalation, 6 hours/day, 5 days/week, 11, 13, 15 weeks	+^a)^	no data for cytotoxicity; statistical significance after 11 and 13 weeks	Donner et al. 1981
chromosomal aberrations, bone marrow	rat, Wistar, ♂, no other data	0, 300 ml/m^3^, inhalation, 6 hours/day, 5 days/week, 15 weeks	–	no data for cytotoxicity	Donner et al. 1981
chromosomal aberrations, bone marrow	rat, albino (no other data), groups of 6 ♂	0, 160 ml/m^3^, inhalation, 4 hours/day, 5 days/week, 4, 10, 16 weeks	+	sum total of gaps, breaks, translocations, ring chromosomes and dicentric chromosomes; statistical significance ≥ 4 weeks	Dobrokhotov and Enikeev 1977
chromosomal aberrations, bone marrow	rat, albino (no other data), groups of 8 ♂	0, 1.4 ± 0.2, 13.3 ± 0.4 ml/m^3^, inhalation, 4 hours/day, 5 days/week, 16 weeks	–		Aristov et al. 1981
chromosomal aberrations, bone marrow	rat, no other data, groups of 5 ♂	12 × 0, 800 mg/kg body weight and day, subcutaneous, no other data	+	no data for cytotoxicity	Dobrokhotov 1972
chromosomal aberrations, bone marrow	rat, albino (no other data), groups of 6 ♂	12 × 0, 1000 mg/kg body weight and day, subcutaneous	+	metaphases with chromosomal aberrations: 11.7%; of these, 60.5% gaps and 39.5% breaks; in the blood: leukocytosis, anaemia; in the bone marrow: cell proliferation and hyperplasia	Lyapkalo 1973
chromosomal aberrations, bone marrow	rat, no other data, groups of 5 ♂	1 × 0, 22, 71, 215 mg/kg body weight (0.025, 0.082, 0.247 ml/kg body weight), intraperitoneal, 6, 24 or 48 hours; 0, 22, 71, 215 mg/kg body weight and day (0.025, 0.082, 0.247 ml/kg body weight), intraperitoneal, 1 per day, 5 days, examined 6 hours after last dose	–	cytotoxicity at 71 mg/kg body weight and above	Litton Bionetics Inc 1983
chromosomal aberrations, bone marrow	mouse, CD 1, groups of 5 ♀/♂	2 × 0, 1720^b)^ mg/kg body weight, oral, at an interval of 24 hours, examined 30 hours after the first dose	–	no data for cytotoxicity; purity: 99%	Gad-El Karim et al. 1984
chromosomal aberrations, bone marrow	mouse, random-bred SHR, ♂ (no other data)	10 × each of 4 different doses of 0.001–0.2 × LD_50_, gavage, no other data	–		Feldt et al. 1985
chromosomal aberrations, bone marrow	rat, Crj: Sprague Dawley, groups of 5 ♂	2 × 0, 109, 218, 435 mg/kg body weight, intraperitoneal, at an interval of 24 hours, examined 30 hours after the first dose	+	statistical significance at 435 mg/kg body weight; cytotoxicity significant ≥ 217.5 mg/kg body weight; increase in pulverized cells, breaks and gaps; no translocations	Roh et al. 1987
micronuclei, bone marrow	mouse, Crl:CD 1, groups of 5 ♀/♂	2 × 0, 1720^b)^ mg/kg body weight, oral, at an interval of 24 hours, examined 30 hours after the first dose	–	no data for cytotoxicity; purity: 99%	Gad-El Karim et al. 1984
micronuclei, bone marrow	mouse, Crl:CD 1, groups of 5 ♂	1 × 0, 860 mg/kg body weight, oral, 30 hours	–	no data for cytotoxicity; purity: 99%	Gad-El Karim et al. 1986
micronuclei, bone marrow	mouse, NMRI and B6C3F1, groups of 5 ♂	2 × 0, 0.12, 0.25, 0.37, 0.5 ml (104, 218, 322, 435 mg)/kg body weight, intraperitoneal, at an interval of 24 hours, examined 30 hours after first injection	+	no data for cytotoxicity; NMRI: positive results ≥ 2 × 0.25 ml/kg body weight; B6C3F1: positive results ≥ 2 × 0.12 ml/kg body weight; LD_50_ (NMRI): 1.64 ml/kg body weight; LD_50_ (B6C3F1): 1.88 ml/kg body weight; purity: 99%	Mohtashamipur et al. 1985
micronuclei, bone marrow	mouse, NMRI, groups of 5 ♂	2 × 0, 0.25, 0.37, 0.5 ml (218, 322, 435 mg)/kg body weight, at an interval of 24 hours, 1 × 0, 0.5 ml (435 mg)/kg body weight, intraperitoneal, examined 30 hours after first injection	+	no data for cytotoxicity; positive results with statistical significance: 1 × 0.5 and 2 × 0.37, 0.5 ml/kg body weight; purity: 99%, vehicle: corn oil	Mohtashamipur et al. 1987
micronuclei, bone marrow	rat, Crj: Sprague Dawley, groups of 5 ♂	2 × 0, 109, 218, 435 mg (0.125, 0.25, 0.5 ml)/kg body weight, intraperitoneal, at an interval of 24 hours, examined 30 hours after first injection	+^c)^	statistical significance only at 218 mg/kg body weight; cytotoxicity with statistical significance ≥ 218 mg/kg body weight; vehicle: olive oil	Roh et al. 1987
micronuclei, blood	mouse, CD 1, groups of 12 ♂	0, 100 ml/m^3^, inhalation (whole body), 6 hours/day, 8 days, examined 18 hours after last dose	–	with/without GSH depletion^d)^	Bird et al. 2010
micronuclei, bone marrow	mouse, Crl:CD 1, 10 ♂	0, 100 ml/m^3^, inhalation (whole body), 6 hours/day, 8 days within a 15-day period (treatment on days 1, 2, 5, 7, 9, 12, 13 and 15); examined 18 hours after last dose	–	PCE/NCE unchanged; purity > 90%	Wetmore et al. 2008
dominant lethal test	mouse, random-bred SHR, ♂, no other data	0.001–0.2 × LD_50_, gavage, 5 weeks, no other data	–	linear dose–response relationship; PCE increased with statistical significance ≥ 200 mg/kg body weight; no other data	Feldt et al. 1985
dominant lethal test	mouse, CD 1, groups of 12 ♂	0, 100, 400 ml/m^3^, inhalation, 6 hours/day, 5 days/week, 8 weeks, mating for 2 weeks with 2 untreated ♀ each per week	–		Litton Bionetics Inc 1983

a) Sister chromatid exchange increased with statistical significance in weeks 11 and 13, but not in week 15.

b) additional information in text: “similar results achieved with higher and lower doses, oral and intraperitoneal administration” (Gad El Karim and Legator, unpublished data)

c) statistical significance only concurrently with toxicity (decrease of PCE/NCE)

d) To study the role of GSH depletion on the toluene-mediated induction of micronuclei, 12 animals were treated intraperitoneally with buthionine sulfoximine doses of 4 mmol/kg body weight twice a day over the entire course of the study.

NCE: normochromatic erythrocytes; PCE: polychromatic erythrocytes; SLRL: test for X-chromosomal recessive lethal mutations (sex-linked recessive lethal test)

##### Combined effect of toluene and benzene

The findings relating to genotoxicity induced by the simultaneous application of toluene and benzene are inconsistent. All studies found benzene to be clearly genotoxic in its pure form, unlike pure toluene, which did not cause genotoxic effects. As a mixture, however, there were signs of both additive and subtractive effects. Simultaneous oral, subcutaneous or intraperitoneal administration of toluene and benzene reduced benzene-induced damage in mice and rats that was determined as SCE (Tice et al. 1982) or micronuclei (Gad-El Karim et al. 1984; Roh et al. 1987; Tunek et al. 1982).

By contrast, the number of micronuclei was higher in mice exposed by inhalation to a mixture (benzene:toluene; 1:1 and 1:2) than in mice exposed only to benzene (Bird et al. 2010; Wetmore et al. 2008).

#### 5.6.3 Summary

In vitro, toluene did not cause mutagenic effects in bacteria and did not induce mutagenic, clastogenic or aneugenic effects in mammalian cells in tests for SCE, micronuclei, chromosomal aberrations and gene mutations in the concentration range that did not lead to marked cytotoxicity. At marked levels of cytotoxicity, a higher incidence of kinetochore-positive and kinetochore-negative micronuclei was induced in the micronucleus test.

In vivo tests for chromosomal aberrations and micronuclei yielded inconsistent results. A number of tests obtained negative results after inhalation, oral and intraperitoneal administration. In several other tests, however, clastogenic effects were observed after subcutaneous and intraperitoneal administration, but either only concurrently with cytotoxicity or without measuring (cyto)toxicity.

No lethal mutations were induced in Drosophila or in mice.

Therefore, toluene has not been found to induce genotoxicity.

## 6 Manifesto (MAK value/classification)

The critical effects induced by toluene are neurotoxic effects, particularly on the CNS. Behavioural toxicity and neurophysiological effects were observed after acute exposure. Chronic exposure is associated with behavioural toxicity and ototoxicity and may lead to an impairment of colour vision.

**MAK value. **The MAK value of 50 ml/m^3^ was derived in 1993 based on the neurotoxic effects in humans (Greim 1996). Later studies, which were described in the supplement from 2002 in summary form (Hartwig and MAK Commission 2021 b), did not provide evidence that chronic effects are induced by toluene in concentrations of about 50 ml/m^3^. The data are derived from the findings of an epidemiological longitudinal study that was carried out in the rotogravure printing industry and take into account also individual estimates of lifelong exposure to toluene. A few recent studies reported that toluene induced effects such as the impairment of cognitive performance, hearing and colour vision at concentrations below 50 ml/m^3^. These studies are all cross-sectional studies; only a few of them took co-factors (for example age, education, etc.) adequately into consideration. It is difficult to determine, or estimate, the extent of chronic exposure as no quantitative estimates of cumulative exposure were available at the time of testing. In some cases, it was not possible to rule out exposure to a mixture of solvents. Extensive, well-controlled experimental studies of short-term effects did not find evidence of behavioural toxicity expressed as a significant decline in neuropsychological test performance after exposure to toluene at concentrations below 50 ml/m^3^ (in some cases also above). PBPK model-based calculations of short-term effects on increasing the choice reaction time predict a 10% reduction in performance after exposure to toluene concentrations < 50 ml/m^3^. These predictions show that there is considerable uncertainty with regard to the modelling of toluene concentrations and the review of test performance in the underlying studies in test persons. Under controlled exposure conditions designed to investigate the effects of concentration peaks on cognitive and neurophysiological end points, effects on neurophysiological parameters, but not on performance tests, were found at peak values of 200 ml/m^3^ and simultaneous physical exertion. Most animal studies that investigated low levels of exposure focused on the effects induced by toluene on a molecular level, but it is unknown whether these results can be compared directly with the findings of studies in humans. Therefore, based on the findings reported by numerous studies in humans, the previous MAK value of 50 ml/m^3^ has been retained.

**Peak limitation. **The critical effect of toluene is neurotoxicity; for this reason, its classification in Peak Limitation Category II has been retained. As peak levels of exposure of 200 ml/m^3^ and simultaneous physical exertion (Yavari et al. 2018) did not have an effect on performance tests, but on neurophysiological parameters, the excursion factor has been lowered to 2.

**Prenatal toxicity. **Up until this point, toluene has been classified in Pregnancy Risk Group C with a MAK value of 50 ml/m^3^.

**Developmental toxicity:**

In the children of mothers who inhaled high concentrations of toluene in the range from 4000 to 12 000 ml/m^3^ or other organic solvents during pregnancy, toluene induced symptoms similar to those of the foetal alcohol syndrome caused by exposure to ethanol. Concentration-dependent embryolethality and delays in foetal growth and skeletal development were observed.

There are no reliable studies available that investigated the exposure of humans in the low dose range.

Prenatal developmental toxicity studies in rats did not detect toxic effects on development after inhalation exposure to concentrations up to 750 ml/m^3^ for 6 to 7 hours a day during gestation. At concentrations of 1000 ml/m^3^ to 3500 ml/m^3^, reduced foetal weights, and delays in growth and foetal skeletal development were induced concurrently with maternal toxicity in the form of decreased body weights. No teratogenicity occurred in this concentration range. A postnatal effect observed in rats at 1200 ml/m^3^ was a delay in incisor eruption. Embryolethality or abortive effects in animals were not induced by toluene up to a concentration of 3500 ml/m^3^ (see Table 1; ATSDR 2017).

In the supplement from 1993 (Greim 1996), NOAECs of 750, 500 and 400 ml/m^3^, respectively, were derived for developmental toxicity in rats, rabbits and mice after inhalation exposure (HRC 1992; Jones and Balster 1997; Klimisch et al. 1992; Roberts et al. 1993). These effects were observed concurrently with maternal toxicity. This resulted in 15-fold, 10-fold and 8-fold margins, respectively, to the MAK val^u^e of 50 ml/m^3^ with the procedure used at the time. In mice and rabbits, these were the highest concentrations tested. By taking the increased respiratory volume at the workplace in comparison with that of the animal at rest (1:2) into consideration, this would result in 8-fold, 5-fold and 4-fold margins, respectively, for the rat, rabbit and mouse after inhalation exposure. A study carried out by Thiel and Chahoud (1997) in rats likewise found that exposure to concentrations of 1000 and 1200 ml/m^3^ led to an 8% reduction in foetal weights on postnatal day 1 concurrently with maternal toxicity in the form of reduced body weights and reduced body weight gains. However, no effects on the body weights of the offspring were noticeable from postnatal day 14 onwards. The NOAEC for prenatal developmental toxicity was 600 ml/m^3^ and the LOAEC was 1000 ml/m^3^, which would be equivalent to a 6-fold and 10-fold margin, respectively, to the MAK value of 50 ml/m^3^ if the increased respiratory volume (1:2) is taken into consideration.

The foetuses of rats given gavage doses of 1250 mg/kg body weight and day on gestation days 16 to 19 were found to have dilated renal pelvises (variation) (Warner et al. 2008). The NOAEL was 750 mg/kg body weight and day (see Table 2). The following toxicokinetic data are taken into consideration for the extrapolation of this NOAEL to a concentration in workplace air: the corresponding species-specific correction value for the rat (1:4), the oral absorption of 100% determined experimentally in rats (Turkall et al. 1991), the body weight (70 kg) and the respiratory volume (10 m^3^) of the person and the 50% absorption by inhalation determined experimentally in humans (Löf et al. 1993). The concentration calculated from this is 691 ml/m^3^ (2626 mg/m^3^), which is equivalent to a 14-fold margin to the MAK value.

**Developmental neurotoxicity:**

Since 2016, a statement on developmental neurotoxicity in the foetus has been required for substances with MAK values derived from neurotoxic effects.

In behavioural tests carried out with rats, no changes were noticeable at exposure levels of 400 to 1500 ml/m^3^. NOAECs of 1200 and 2000 ml/m^3^ were derived from the findings of developmental neurotoxicity studies in rats; these represent adequate 12-fold and 20-fold margins, respectively, to the MAK value, taking into consideration the increased respiratory volume (1:2). The behavioural studies in mice had considerable methodological shortcomings and have not been included in the evaluation of developmental neurotoxicity (see Table 3).

Overall, the NOAEC for developmental toxicity is lower than that for developmental neurotoxicity. Toluene induced slight unspecific delays in development concurrently with maternal toxicity, but no teratogenicity in animals up to 3500 ml/m^3^. The most sensitive end point is the decrease in perinatal body weights. On the basis of the toxicokinetic data for the absorption of toluene after inhalation exposure and the resulting blood concentrations, marked differences between humans and test animals were not found. In rats, only very slight effects on the body weights of the offspring were observed on postnatal day 1 at the two high concentrations; these were reversible after 2 weeks and were observed only concurrently with maternal toxicity (Thiel and Chahoud 1997). Taking into consideration the increased respiratory volume (1:2), there is a 10-fold margin between the LOAEC of 1000 ml/m^3^ determined for these minor and reversible effects and the MAK value. Toluene therefore remains classified in Pregnancy Risk Group C in spite of the narrow 6-fold margin between the NOAEC for developmental toxicity and the MAK value of 50 ml/m^3^.

**Germ cell mutagenicity. **Toluene induced SCE in male rotogravure printing workers who were exposed by inhalation to high toluene concentrations of 200 to 300 ml/m^3^ (< 0.3% benzene) for at least 16 years with additional exposure through the skin. Clastogenic effects were reported by several studies that investigated exposure to toluene within a mixture of solvents. Toluene did not induce clastogenic effects in male test persons after controlled exposure at the level of the MAK value of 50 ml/m^3^.

In vitro, toluene did not cause mutagenic effects in bacteria and did not induce mutagenic, clastogenic or aneugenic effects in mammalian cells in tests for SCE, micronuclei, chromosomal aberrations and gene mutations in the concentration range that did not lead to marked cytotoxicity. At marked levels of cytotoxicity, a higher incidence of kinetochore-positive and kinetochore-negative micronuclei was induced in the micronucleus test. In vivo tests for chromosomal aberrations and micronuclei yielded inconsistent results. A number of tests obtained negative results after inhalation, oral and intraperitoneal administration. In several other tests, however, clastogenic effects were observed after subcutaneous and intraperitoneal administration, but either only concurrently with cytotoxicity or without measuring (cyto)toxicity. No lethal mutations were induced in germ cells in Drosophila or in mice.

Therefore, toluene is not regarded as genotoxic. The data available do not suggest germ cell mutagenicity. For this reason, toluene has not been classified in a category for germ cell mutagenicity.

**Absorption through the skin. **The supplement from 1998 (Hartwig and MAK Commission 2021 a) already established the evidence base for designating toluene with an “H” (for substances which can be absorbed through the skin in toxicologically relevant amounts) at a MAK value of 50 ml/m^3^. In test persons, exposure to liquid toluene under standard conditions led to absorption through the skin in an amount equal to that taken up by the lungs after exposure at the level of the MAK value. For this reason, the “H” designation has been retained.

**Sensitization. **There are no data for sensitizing effects in humans and no positive results from animal studies or in vitro studies. For this reason, toluene has not been designated with “Sh” or “Sa” (for substances which cause sensitization of the skin or airways).
